# Spatiotemporal modulation of nitric oxide and Notch signaling by hemodynamic-responsive Trpv4 is essential for ventricle regeneration

**DOI:** 10.1007/s00018-023-05092-0

**Published:** 2024-01-27

**Authors:** Chunxiao Yu, Xueyu Li, Jinmin Ma, Shuzhang Liang, Yan Zhao, Qi Li, Ruilin Zhang

**Affiliations:** 1 TaiKang Medical School, School of Basic Medical Sciences, Wuhan University, Wuhan, 430071 China; 2 School of Life Sciences, Fudan University, Shanghai, 200433 China; 3 Medical Frontier Innovation Research Center, The First Hospital of Lanzhou University, The First Clinical Medical College of Lanzhou University, Lanzhou, 730000 China; 4 Institute of Myocardial Injury and Repair, Wuhan University, Wuhan, 430071 China; 5 Hubei Provincial Key Laboratory of Developmentally Originated Disease, Wuhan, 430071 China

**Keywords:** Heart regeneration, Hemodynamics, Trpv4, NO signal, TGF-β

## Abstract

Zebrafish have a remarkable ability to regenerate injured hearts. Altered hemodynamic forces after larval ventricle ablation activate the endocardial Klf2a*-*Notch signaling cascade to direct zebrafish cardiac regeneration. However, how the heart perceives blood flow changes and initiates signaling pathways promoting regeneration is not fully understood. The present study demonstrated that the mechanosensitive channel Trpv4 sensed the altered hemodynamic forces in injured hearts and its expression was regulated by blood flow. In addition to mediating the endocardial Klf2a-Notch signal cascade around the atrioventricular canal (AVC), we discovered that Trpv4 regulated nitric oxide (NO) signaling in the bulbus arteriosus (BA). Further experiments indicated that Notch signaling primarily acted at the early stage of regeneration, and the major role of NO signaling was at the late stage and through TGF-β pathway. Overall, our findings revealed that mechanosensitive channels perceived the changes in hemodynamics after ventricle injury, and provide novel insights into the temporal and spatial coordination of multiple signaling pathways regulating heart regeneration.

**Supplementary Information:**

The online version contains supplementary material available at 10.1007/s00018-023-05092-0.

## Introduction

Myocardial infarction (MI) is one of the most devastating diseases worldwide [1], primarily due to the unsatisfactory replenishment of lost cardiomyocytes (CMs) in adult mammalian hearts [2]. In contrast, zebrafish hearts possess a strong regenerative ability after injury via the dedifferentiation, proliferation, and migration of pre-existing CMs [3, 4]. Multiple coordinating signaling pathways regulate these cellular processes during regeneration [5], and the mechanisms of pathway activation are under extensive investigation. Our recent report demonstrated alterations in intracardiac blood flow and hemodynamic shear stress after larval ventricle ablation, which were essential for activation of the endocardial flow-sensitive transcription factor Klf2 and Notch signaling [6, 7]. We further showed that primary cilia were critical for the perception and transmission of mechanical shear stress signals during ventricle regeneration, but the involvement and functions of other mechanosensors remain to be explored.

Transient receptor potential vanilloid 4 (TRPV4) is a well-known mechanosensitive ion channel that plays vital roles in cardiovascular development and diseases [8, 9]. Activation of TRPV4 is involved in hypoxia/reoxygenation injury and congestive cardiac failure [10], and the TRPV4 antagonist HC-067047 significantly reduced the myocardial infarction area and improved cardiac function in an ischemia–reperfusion (I/R) model [11]. Heckel et al. discovered that oscillatory flow modulated mechanosensitive *klf2a* expression via Trpv4 and Trpp2 during heart valve development [12]. We previously showed that *trpv4* deficiency blocked heart regeneration by suppressing the Klf2a-Notch signaling cascade [6]. However, the detailed functions of Trpv4 in regulating cardiac regeneration and whether signaling pathways other than Notch are involved in regeneration warrant further investigation.

TRPV4 affects vasodilation by regulating the synthesis of nitric oxide (NO) in mesenteric arteries in mice [13]. NO is a relaxation factor that is widely distributed in cardiovascular tissues in mammals, and it regulates vasodilation, platelet aggregation, smooth muscle cell proliferation and myocardial contractile function [14–16]. NO also plays an important role in the zebrafish cardiovascular system. NO modulated myocardial performance in fish hearts [17]. The blood flow-dependent Klf2a-NO signaling cascade was required for stabilization of hematopoietic stem cell programming in zebrafish embryos [18]. Despite the importance of NO signaling in multiple biological processes, its role in heart regeneration remains largely unexplored.

The present study investigated the underlying molecular mechanisms of the sensation and transmission of mechanical hemodynamic signals during cardiac regeneration. We used in vivo live imaging to monitor the dynamic NO level in the injured zebrafish larval hearts and confirmed that blood flow regulated NO production. We discovered that the mechanosensitive channel Trpv4 sensed and transduced the altered hemodynamic flow to modulate NO and Notch signaling. Further studies demonstrated that NO and Notch signaling regulated ventricle regeneration at different stages and locations, and NO signaling exerted its functions through secretory TGF-β pathway. Taken together, our results revealed a critical Trpv4-mediated biomechanical signal cascade via spatiotemporal modulation of NO and Notch signaling during zebrafish ventricle regeneration.

## Materials and methods

### Zebrafish husbandry

Zebrafish were raised and maintained under standard conditions. All experiments were performed according to institutional and national animal welfare guidelines. We used the following transgenic lines: *Tg(vmhc:mCherry-NTR)*, *Tg(kdrl:mCherry-ras)*, *Tg(kdrl:eGFP)*, *Tg(amhc:CreERT2; β-act2:RSG)*, *Tg(myl7:mAG-zGeminin)*, *Tg(myl7:H2B-eGFP)*, *Tg(myl7:eGFP-ras)*, *Tg(tp1:d2GFP)*. E3 water with 0.003% PTU (1-phenyl-2-thiourea, Sigma) was used for embryos and larvae over 24 hpf to prevent pigmentation.

### Generation of mutant and crispant zebrafish

*Trpv4*^−/−^ mutants were generated using the CRISPR/Cas9 technique [19]. sgRNA target sites were identified via CRISPRscan website (www.crisprscan.org). The sgRNAs were in vitro transcribed from PCR products amplified with specific forward primer and a universal reverse primer (Supplementary Table 1), and co-injected with 50 ng/μl Cas9 protein (New England Biolabs) into embryos at the one-cell stage. Positive founders were mated with wild-type fish to obtain F1 generation, and F1 heterozygous zebrafish were intercrossed to generate F2 homozygous mutants. *nos1*, *nos2a* and *nos2b* crispants were generated using a similar protocol, except two sgRNAs for each gene were simultaneously co-injected with Cas9 protein into embryos at the one-cell stage and the phenotypes were observed in F0 generation.

### Chemical treatment

*Tg(vmhc:mCherry-NTR)* larvae were treated with 6.5 mM MTZ (Metronidazole, Sigma) or 0.2% DMSO (dimethyl sulfoxide, Fisher Scientific) in E3 water for 4 h at 72 hpf as previously described [4]. After washed with fresh E3 water for several times, larvae were treated with following chemicals for indicating time periods: 2 mM L-NMMA (N^G^-monomethyl-L-arginine monoacetate, MCE), 10 mM BDM (2,3-Butanedione monoxime, Sigma), 1.8 mM tricaine (3-aminobenzoic acid ethyl ester, Sigma), 100 μM DAPT (N-[N-(3,5-Difluorophenacetyl)-L-alanyl]-S-phenylglycine t-butyl ester, Sigma), 2.5 μM HC-067047 (MCE), 10 μM 4α-pdd (4α-Phorbol 12,13-didecanoate, Sigma), 1 mM SNP (sodium nitroprusside dihydrate, Sigma) or 500 μM SRI-011381 (MCE).

### In situ hybridization

Whole-mount in situ hybridization was performed as previously described [7, 20], including following probes: *trpv4*, *klf2a*, *snail1a*, *twist1a*, *vimentin*, *nkx2.5*, *hand2*, *gata4*, *tgfb1a* and *tgfb1b*. Larvae were analyzed under a Nikon SMZ18 stereo microscope.

### Immunofluorescence

Immunofluorescence staining on dissected larval hearts or whole-mount larvae was performed as previously described [4]. The primary antibodies used were anti-Trpv4 (rabbit; AtaGenix laboratories, Wuhan, China), anti-phospho-histone H3 (rabbit; Merck Millipore, 06570), anti-phospho-Smad3 (rabbit; Abcam, 52,903) and anti-MHC (mouse; DSHB, MF20). The secondary antibodies used were Alexa Fluor 488 goat anti-mouse IgG, Alexa Fluor 488 goat anti-rabbit IgG, Alexa Fluor 555 goat anti-mouse IgG, and Alexa Fluor 555 goat anti-rabbit IgG from Invitrogen. Fluorescence images were obtained using a Leica SP8 confocal microscope.

### Morpholino injection

Morpholino injection was performed as previously described [21]. The morpholino against *tnnt2a* (5′-CATGTTTGCTCTGATCTGACACGCA-3′) was purchased from GeneTools (Philomath, OR, USA). 1 ng *tnnt2a* MO was injected into embryos at the one-cell stage, all injected embryos were used for indicating experiments at specific stages.

### Quantitative real-time PCR

Total RNA was extracted from 30 larvae or 250 dissected hearts in TRIeasy™ reagent (Yeasen, Shanghai, China) with Freezing grinder (Wonbio). cDNA was synthesized with a ReverTra Ace qPCR RT Kit (TOYOBO), quantified with Taq Pro Universal SYBR qPCR Master Mix (Vazyme Biotech Co., Ltd) and normalized by β-actin as internal control. Sequences of primers used were summarized in Supplementary Table 1.

### DAF-FM DA staining

Larvae were incubated in E3 water containing 5 μM DAF-FM DA (Invitrogen, D23844) for 90 min in the dark at 28 °C. After incubation, larvae were rinsed in E3 water for several times and waited for 30 min before imaging. Fluorescence images were obtained using a Leica SP8 confocal microscope.

### Quantification and statistical analysis

The regeneration ratio was calculated as the numbers of recovered larvae over the total numbers of injured larvae. Recovered larvae had restored ventricular size and contractile functions close to control hearts (> 90%) as previously described [4], and the numbers were counted by blinded assessments. Values were presented as mean ± s.e.m. Statistical significance was defined as a threshold of *P* < 0.05 determined by Student’s t-test between two groups, ANOVA analysis between more than two groups or Binomial test in quantification of the percentage of recovered hearts.

## Results

### Inhibition of NO synthesis impedes zebrafish ventricle regeneration

**Fig. 1:**
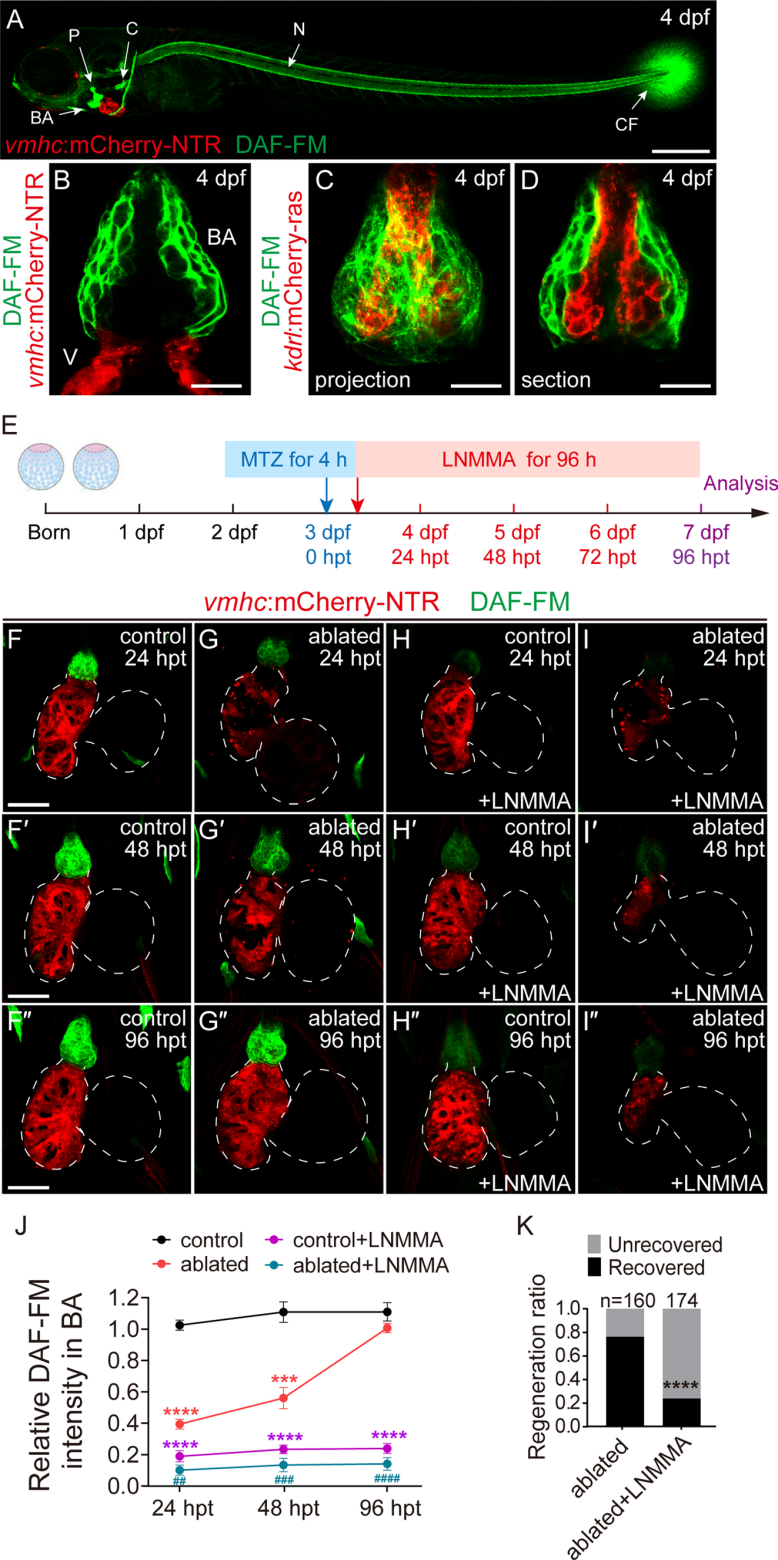
Inhibition of nitric oxide synthesis impedes zebrafish ventricle regeneration. **A** DAF-FM DA staining showed that NO (green) was primarily present in the bulbus arteriosus (BA), cleithrum (C), pharyngeal jaw bone (P), notochord (N) and caudal fin (CF) in larvae at 4 dpf. Scale bar, 150 μm. **B**–**D** DAF-FM DA staining of *Tg(vmhc:mCherry-NTR)* or *Tg(kdrl:mCherry-ras)* hearts at 4 dpf showed that NO was enriched in the smooth muscle layer of the BA. Scale bars, 10 μm. **E** Schematic timeline diagram of MTZ treatment to induce ventricle ablation and L-NMMA treatment to inhibit NO production. **F**–**I**″ The NO level was significantly decreased in ablated hearts at 24 hpt and gradually increased to a level comparable to control hearts at 96 hpt (**F**–**G**″). L-NMMA treatment dramatically reduced NO level in control and ablated hearts (**H**–**I**″). Dashed lines outline the hearts. Scale bars, 50 μm. **J** Quantification of relative DAF-FM DA intensity of BA in control or ablated hearts with or without L-NMMA treatment. N = 5 for each group. Mean + s.e.m. ANOVA analysis, ***P < 0.001, ****P < 0.0001 as compared with control group; ^*##*^*P* < 0.01, ^*###*^*P* < 0.001, ^*####*^*P* < 0.0001 as compared with ablated group. **K** Quantification of the heart recovery rate in the ablated and ablated + L-NMMA-treated groups at 96 hpt. The numbers of larvae analyzed for each condition are indicated. Binomial test, ***P < 0.001, ****P < 0.0001. *dpf* days post fertilization, *hpt* hours post treatment, *V* ventricle, *BA* bulbus arteriosus, *NO* nitric oxide

NO signaling mediates cardiovascular development in mammals [22], but the role of NO in heart regeneration is not clear. To investigate this issue, we used the fluorescent probe diaminofluorescein-FM diacetate (DAF-FM DA) [23] to detect intracellular NO in live zebrafish larvae. At 4 days post-fertilization (dpf), a strong fluorescence signal was primarily present in the bulbus arteriosus (BA), cleithrum (C), pharyngeal jaw bone (P), notochord (N) and caudal fin (CF) (Fig. 1A), as previously reported [24]. To further examine the tissue distribution of NO in larval hearts, the myocardial reporter line *Tg(vmhc:mCherry-NTR)* and endothelial reporter line *Tg(kdrl:mCherry-ras)* were used with DAF-FM DA staining. The fluorescence was not present in the myocardial or endothelial layer of the hearts but localized primarily in the smooth muscle layer of the BA (Fig. 1B–D).

We examined the role of NO in heart regeneration using the cardiac ventricle ablation and regeneration line *Tg(vmhc:mCherry-NTR)* [4] (Fig. 1E). The control group exhibited a strong and stable NO signal in the BA during the entire period (Fig. 1F–F″, J). Metronidazole (MTZ) treatment at 3 dpf for 4 h (h) severely ablated the ventricle and significantly decreased DAF-FM DA fluorescence in the BA at 24 h post-treatment (hpt). The levels gradually increased to a level comparable to the control group at 96 hpt (Fig. 1G–G″, J), when the injured hearts completed regeneration with restored ventricular fluorescence and contractile functions as previously reported [4]. MTZ treatment on *Tg(myl7:mCherry)* larvae, which did not express the key converting enzyme nitroreductase (NTR), had no effect on NO level (Supplementary Fig. 1A, B).

To validate the specificity of DAF-FM DA staining, we used CRISPR/Cas9 genome editing technique to generate F0 crispants for the three zebrafish NO synthases, *nos1*, *nos2a* and *nos2b*. Although the knockout efficiencies were similar (Supplementary Fig. 2A–F), only *nos1* and *nos2b* crispants, but not *nos2a* crispants, showed reduced/absent DAF-FM DA staining in the BA, notochord and caudal fin (Supplementary Fig. 2G–I). The expression levels of *nos1* and *nos2b* in ablated hearts also showed a similar trend that first decreased and then recovered after ventricle ablation (Supplementary Fig. 2 J, K). Since the *nos1* and *nos2b* crispants exhibited pericardiac edema and abnormal heart development (Supplementary Fig. 2H), we then used an NO synthase inhibitor, N^G^-monomethyl-L-arginine monoacetate (L-NMMA) [25], to temporarily inhibit NO production. L-NMMA treatment reduced DAF-FM DA fluorescence in the control and ablated hearts (Fig. 1H–J). This inhibition of NO synthesis dramatically impeded ventricle regeneration. The heart recovery ratio (regenerated hearts/total ablated hearts) at 96 hpt dropped to 24% in the L-NMMA-treated ablated group (N = 174), which was significantly lower than the 78% recovery rate in the ablated group without treatment (N = 160) (Fig. 1K).

### Reduced blood flow suppresses NO production in the BA

**Fig. 2:**
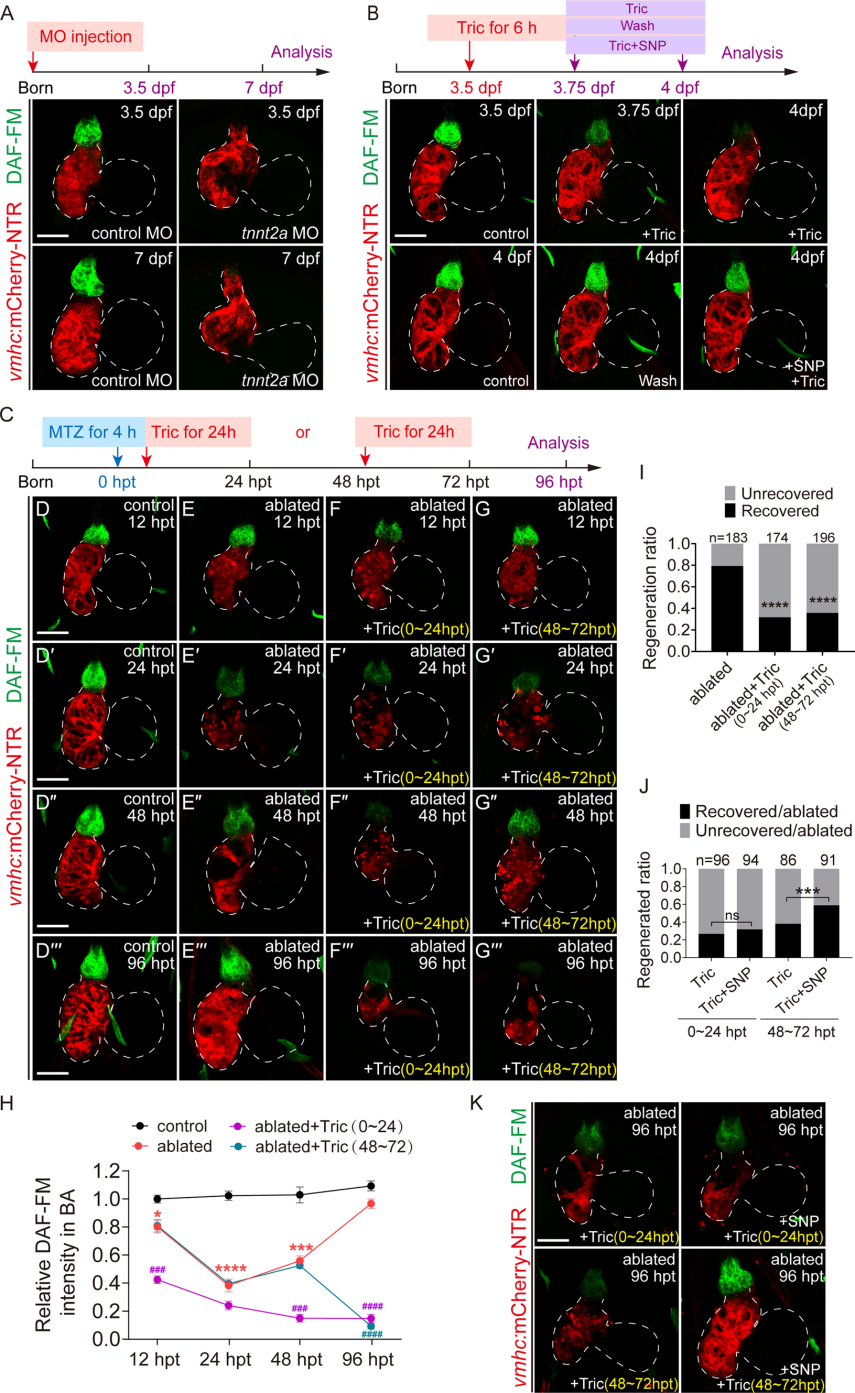
Reduced blood flow suppresses NO production in the BA. **A**, **B** DAF-FM DA staining of *Tg(vmhc:mCherry-NTR)* hearts showed that reduced blood flow via *tnnt2a* MO injection or tricaine treatment markedly suppressed NO production. SNP treatment could supply NO. **C**–**G**′′′ DAF-FM DA staining showed that tricaine treatment for 0–24 or 48–72 hpt significantly reduced NO level in ablated hearts at 96 hpt. **H** Quantification of relative DAF-FM DA intensity of BA in the control, ablated, ablated + Tric-treated (0–24 hpt) and ablated + Tric-treated (48–72 hpt) groups. N = 5 for each group. Mean + s.e.m. ANOVA analysis, *P < 0.05, ***P < 0.001, ****P < 0.0001 as compared with control group; ^*###*^*P* < 0.001, ^*####*^*P* < 0.0001 as compared with ablated group. **I** Quantification of the heart recovery rate in the ablated, ablated + Tric-treated (0–24 hpt) and ablated + Tric-treated (48–72 hpt) groups at 96 hpt. The numbers of larvae analyzed for each condition are indicated. Binomial test, ****P < 0.0001. **J** Quantification of the heart recovery rate in ablated groups at 96 hpt treated with tricaine for 0–24 or 48–72 hpt, with or without co-treatment of SNP. The numbers of larvae analyzed for each condition are indicated. Binomial test; ns, not significant; ***P < 0.001. **K** DAF-FM DA staining of ablated hearts at 96 hpt treated with tricaine for 0–24 or 48–72 hpt, with or without co-treatment of the NO donor SNP. Scale bars, 50 μm. Dashed lines outline the hearts. *dpf* days post fertilization, *hpt* hours post treatment, *NO* nitric oxide, *MO* morpholino, *Tric* tricaine, *SNP* sodium nitroprusside dihydrate

Galvez-Santisteban et al. recently demonstrated increased levels of oscillatory fluctuations of anterograde and retrograde blood flow in injured hearts, which were essential for ventricle regeneration [6]. To investigate whether hemodynamic force regulated NO production, we injected *tnnt2a* morpholino (MO) at the one-cell stage to knock down *cardiac troponin T* expression and abolish heart contraction and blood flow [21]. Injection of *tnnt2a* MO, but not control MO, completely inhibited the synthesis of NO in the BA (Fig. 2A). Because *tnnt2a* knockdown disturbed zebrafish cardiac development, we also treated the larvae with two anesthetics, tricaine and 2,3-butanedione monoxime (BDM), for a short period to temporarily reduce blood flow [7]. Tricaine or BDM treatment significantly suppressed NO production in the BA, which recovered after anesthetic removal (Fig. 2B, Supplementary Fig. 1C–E).

We treated ablated larvae with tricaine for two time periods to observe the effects of reduced blood flow on NO production during ventricle regeneration (Fig. 2C). We previously showed that blood flow changes during 0–24 hpt were important for ventricle regeneration [7]. Tricaine treatment in the ablated group for 0–24 hpt significantly inhibited NO production (Fig. 2D–F′′′, H) and ventricle regeneration (Fig. 2I). Quantification of the heart recovery ratio at 96 hpt showed that the percentage of recovered larvae dropped from 79% (N = 183) to 31% (N = 174) upon tricaine treatment. We also treated ablated larvae with tricaine when the NO signal gradually increases during regeneration (48–72 hpt) and found a similar inhibitory effect on NO production (Fig. 2G–H) and ventricle regeneration (Fig. 2I). The heart recovery ratio was 36% (N = 196).

Sodium nitroprusside dihydrate (SNP) is an NO donor that can supply NO [26]. SNP treatment increased NO production in the hearts treated with tricaine (Fig. 2B, Supplementary Fig. 1E). Ablated larvae were treated with SNP and tricaine at different time periods. NO replenishment by SNP for 0–24 hpt had no effect on regeneration ratio at 96 hpt that was inhibited by tricaine treatment, however, NO replenishment by SNP for 48–72 hpt significantly increased regeneration ratio from 38% (N = 86) to 58% (N = 91) (Fig. 2J, K). Overall, these results suggested that hemodynamic force regulates NO production in developing and regenerating zebrafish hearts, and NO signaling plays an essential role in the ventricle regeneration.

### Hemodynamic-responsive Trpv4 modulates NO and Notch signaling during ventricle regeneration

**Fig. 3:**
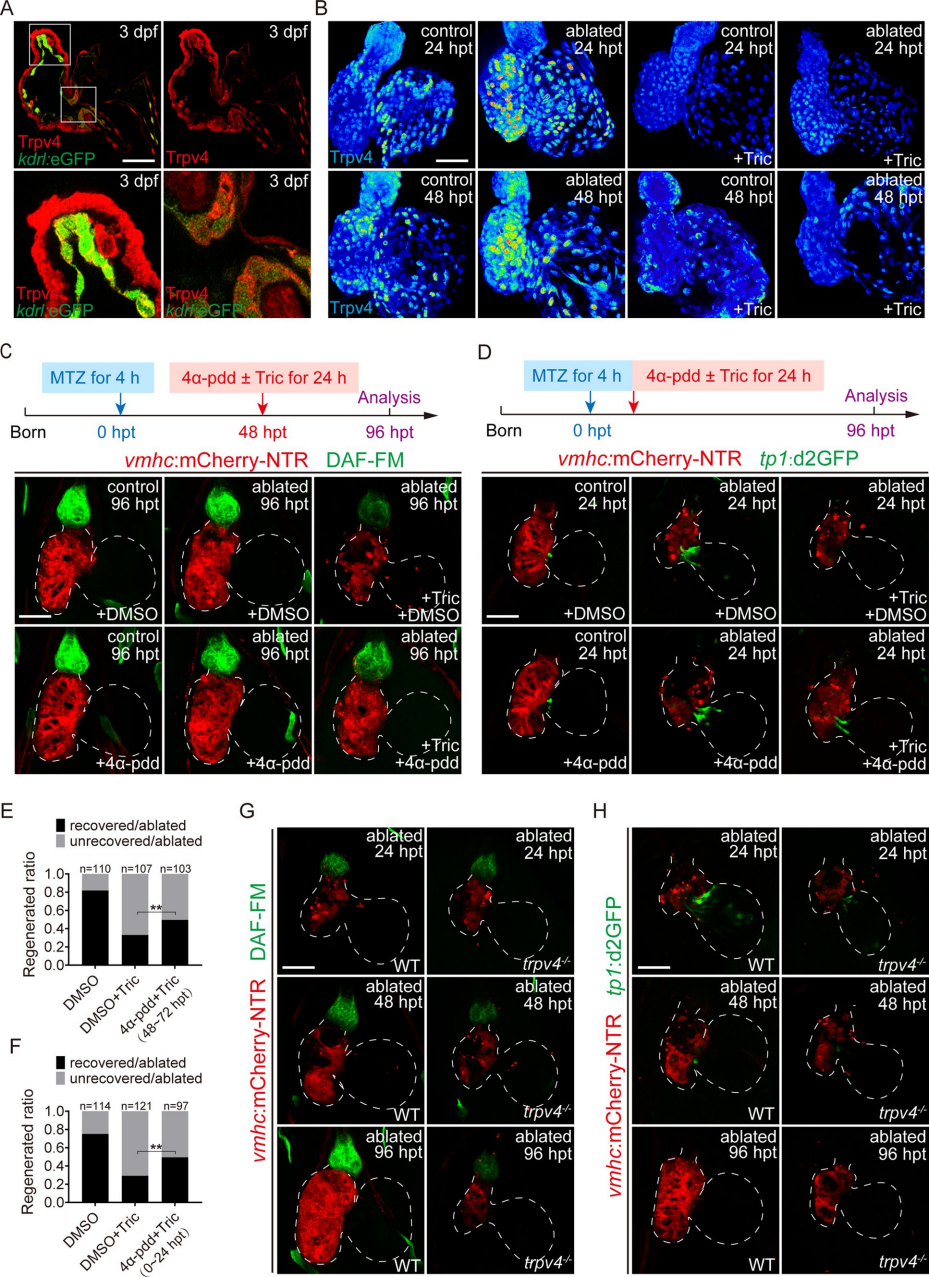
Hemodynamic-responsive Trpv4 modulates NO and Notch signaling during ventricle regeneration. **A** Trpv4 immunostaining (red) in *Tg(kdrl:eGFP)* larvae at 3 dpf showed ubiquitous expression of Trpv4 in the hearts. Bottom panels, enlargement of boxed areas of the bulbus arteriosus (BA) and atrioventricular canal (AVC). **B** Trpv4 immunostaining (intensity gradient) indicated that Trpv4 upregulation after ventricle ablation was blocked in tricaine-treated hearts at 24 and 48 hpt. **C** DAF-FM DA staining of *Tg(vmhc:mCherry-NTR)* larvae showed that Trpv4 agonist 4α-pdd treatment for 48–72 hpt partially rescued the NO signal which was suppressed by tricaine in ablated hearts. **D** Confocal images of *Tg(vmhc:mCherry-NTR; tp1:d2GFP)* hearts showed that Trpv4 agonist 4α-pdd treatment for 0–24 hpt partially rescued Notch signaling which was suppressed by tricaine in ablated hearts. **E**, **F** Quantification of the heart recovery rate in ablated groups treated with DMSO, DMSO + Tric, 4α-pdd + Tric (48–72 hpt) or 4α-pdd + Tric (0–24 hpt) at 96 hpt. The numbers of larvae analyzed for each condition are indicated. Binomial test, **P < 0.01. **G** NO production in ablated hearts was significantly inhibited in *trpv4*^*−/−*^ mutants. **H** Notch signaling activation in ablated hearts was significantly inhibited in *trpv4*^*−/−*^ mutants. Scale bars, 50 μm. Dashed lines outline the hearts. *dpf* days post fertilization, *hpt* hours post treatment, *NO* nitric oxide, *Tric* tricaine

How does hemodynamic force regulate NO signaling? Mechanosensitive channels on cell membranes have been extensively studied, and TRPV4 plays important roles in cardiac development and regeneration [6, 27]. Marziano et al*.* confirmed that TRPV4 mediated vasodilatation of the mesenteric artery by regulating NO synthesis in mice [13]. We performed immunofluorescence staining of Trpv4 in endothelial reporter line *Tg(kdrl:eGFP)* at 3 dpf and observed ubiquitous expression in the endothelium and myocardium of zebrafish hearts, including endothelial cells of the BA and atrioventricular canal (AVC) (Fig. 3A). The expression level of Trpv4 was dramatically increased in ablated hearts of *Tg(vmhc:mCherry-NTR)* larvae at 24 hpt and extended to the BA at 48 hpt, but this increase was abolished by blood flow reduction induced by post-ablation tricaine treatment (Fig. 3B).

We examined whether activation of Trpv4 reversed the inhibitory effect of reduced hemodynamic force on NO production. Because the NO signal in ablated hearts began to recover at 48 hpt, tricaine was added at 48 hpt for 24 h to reduce blood flow with the Trpv4 agonist 4α-phorbol 12,13-didecanoate (4α-pdd) (Fig. 3C). Our results showed that 4α-pdd treatment partially restored DAF-FM DA fluorescence which was suppressed by tricaine treatment in ablated hearts, but the addition of 4α-pdd alone had no effect on the NO signal in the control or ablated groups. The regeneration ratio that was reduced by tricaine in ablated hearts was increased with 4α-pdd treatment (48%, N = 103 vs. 29%, N = 107) (Fig. 3E). Our previous study showed that early endocardial Notch activation in the AVC was vital for cardiac regeneration in zebrafish and was regulated by hemodynamic alteration as well [7]. We used Notch reporter line *Tg(tp1:d2GFP)* and added tricaine and 4α-pdd immediately after ablation for 24 h. The 4α-pdd treatment ameliorated the impediment of Notch activation by tricaine and the regeneration ratio was increased (49%, N = 97 vs. 29%, N = 121), but the addition of 4α-pdd alone had no effect on Notch expression in the control or ablated group (Fig. 3D, F).

To further examine the role of Trpv4 in the regulation of NO production during ventricle regeneration, we generated a new allele of *trpv4*^*−/−*^ mutants using the CRISPR/Cas9 technique, which had a 5-bp deletion that resulted in premature translation termination and displayed similar phenotypes as previously reported allele [6]. The *trpv4* mutants did not show obvious morphological abnormalities during larval development and the heart rate was not different from wild-type larvae (Supplementary Fig. 3). We found that NO production was greatly inhibited in the BA of ablated *trpv4*^*−/−*^ mutant larvae at 96 hpt (Fig. 3G), whereas Notch signaling could not be activated in the AVC of ablated *trpv4*^*−/−*^ mutant larvae at 24 hpt (Fig. 3H). Overall, our findings revealed that blood flow affected the mechanosensitive channel Trpv4, which modulated NO signaling in the BA and Notch signaling in the AVC during ventricle regeneration.

### Temporal inhibition of Trpv4, Notch and NO signaling differentially affects ventricle regeneration

**Fig. 4:**
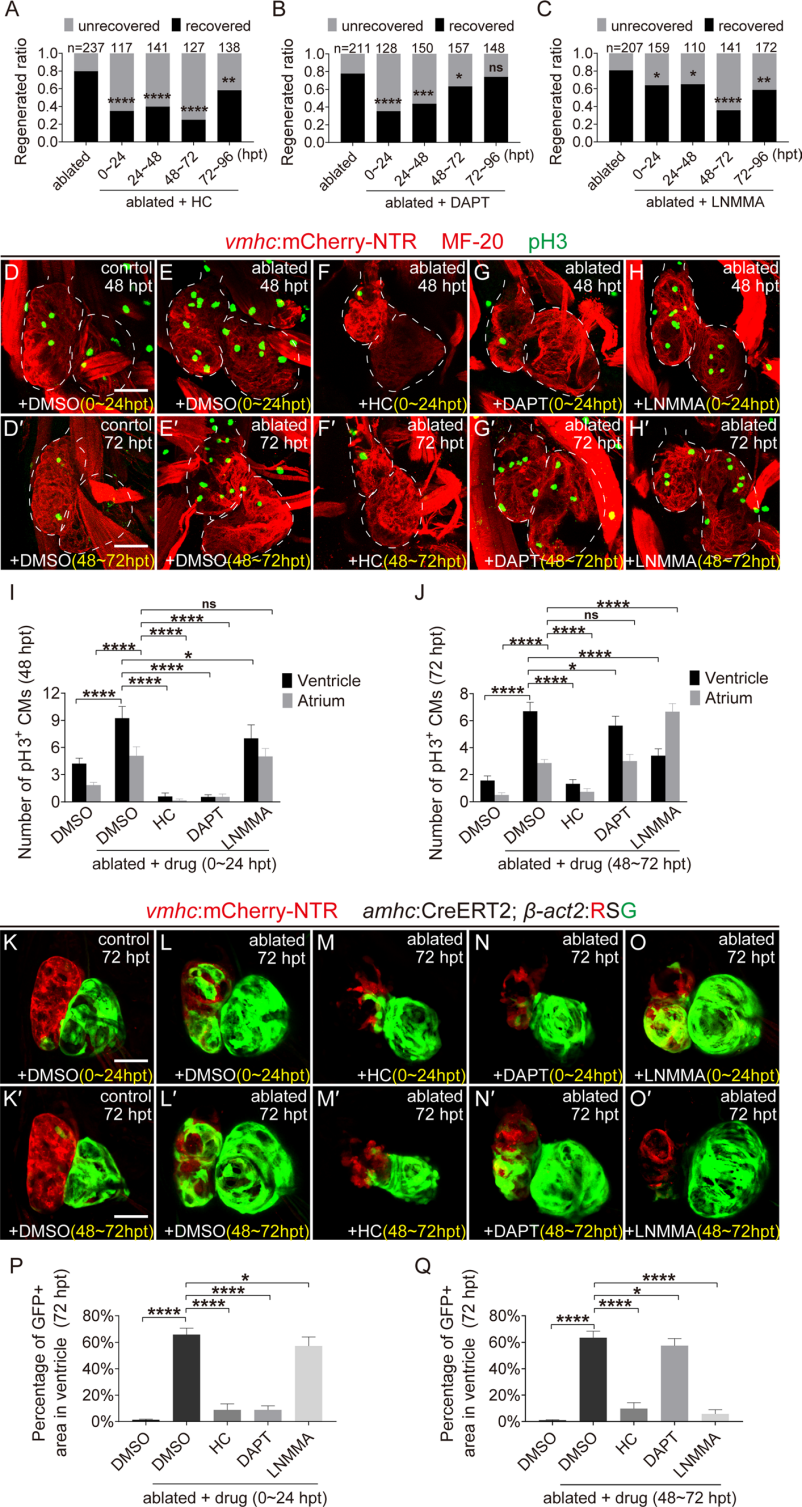
Temporal inhibition of Trpv4, Notch and NO signaling differentially affects ventricle regeneration. **A**–**C** Quantification of the heart recovery rate at 96 hpt in ablated groups treated with HC-067047 (TRPV4 antagonist), DAPT (Notch signaling inhibitor) or L-NMMA (NO synthase inhibitor) at different stages. The numbers of larvae analyzed for each condition are indicated. Binomial test; ns, not significant; *P < 0.05, **P < 0.01, ***P < 0.001, ****P < 0.0001. **D**–**H**′ Immunostaining of anti-phospho-histone H3 (pH3, green) and anti-myosin heavy chain (MF-20, red) in the hearts of control and ablated groups at 48 or 72 hpt with DMSO, HC-067047, DAPT or L-NMMA treatment for 0–24 or 48–72 hpt. **I**, **J** Quantification of pH3^+^ cardiomyocyte numbers in control and ablated hearts treated with DMSO, HC-067047, DAPT or L-NMMA for 0–24 or 48–72 hpt. N = 11 for each group. Mean + s.e.m. ANOVA analysis; ns, not significant; *P < 0.05, ****P < 0.0001. **K**–**O**′ Confocal images of *Tg(vmhc:mCherry-NTR; amhc:CreERT2; β-act2:RSG)* control and ablated hearts at 72 hpt with DMSO, HC-067047, DAPT or L-NMMA treatment for 0–24 or 48–72 hpt. **P**, **Q** Quantification of the percentages of GFP-positive area in control or ablated ventricles with DMSO, HC-067047, DAPT or L-NMMA treatment for 0–24 or 48–72 hpt. N = 10 for each group. Mean + s.e.m. ANOVA analysis; *P < 0.05, ****P < 0.0001. Scale bars, 50 μm. Dashed lines outline the hearts. *hpt* hours post treatment, *HC* HC-067047

We previously showed Trpv4 deficiency in *trpv4*^*−/−*^ mutants impeded early activation of Klf2-Notch signaling and resulted in reduced CM proliferation and heart regeneration (Supplementary Fig. 4) [6]. Pharmacological inhibition of Trpv4 with antagonist HC-067047 [28] at 0–24 hpt displayed similar results (Supplementary Fig. 5). To examine the exact roles of Trpv4, Notch and NO signaling during zebrafish ventricle regeneration, we performed small molecule inhibitor treatment for different time periods and quantified the regeneration ratio at 96 hpt. Treatment with the Trpv4 antagonist HC-067047 at 0–24, 24–48, and 48–72 hpt dramatically reduced the percentages of recovered hearts from 79% in the control ablated group (N = 237) to 34% (N = 117), 39% (N = 141), and 25% (N = 127), respectively, and treatment at 72–96 hpt showed a lower degree of reduction to 58% (N = 138) (Fig. 4A). Inhibition of Notch signaling significantly reduced the regeneration ratio after N-[N-(3,5-Difluorophenacetyl)-L-alanyl]-S-phenylglycine t-butyl ester (DAPT) treatment at 0–24 hpt (35%, N = 128) or 24–48 hpt (43%, N = 150). DAPT treatment at 48–72 hpt only slightly reduced the regeneration ratio (63%, N = 157), and treatment at 72–96 hpt did not obviously change the regeneration ratio (74%, N = 148) (Fig. 4B). Inhibition of NO synthesis using L-NMMA showed a different pattern. L-NMMA treatment at 0–24 and 24–48 hpt slightly reduced the percentages of recovered hearts (63%, N = 159, and 65%, N = 110, respectively), but treatment at 48–72 hpt dramatically reduced the regeneration ratio (35%, N = 141). Treatment at 72–96 hpt produced a lower degree of reduction (58%, N = 172) (Fig. 4C). Treatment with HC-067047, DAPT or LNMMA for 3–7 dpf (0–96 hpt) did not show any toxic effect on heart development in the non-ablated group (Supplementary Fig. 6).

In addition to the regeneration ratio, we also examined CM proliferation after inhibitor treatment using phospho-histone H3 (pH3) immunofluorescence and counterstaining CM with myosin heavy chain antibody MF20. The numbers of pH3^+^ CMs in the ventricle and atrium increased significantly 48 h after heart ablation (ventricle 9.2 ± 4.7, atrium 5.0 ± 3.6, N = 13) (Fig. 4D, E, I), which was similar to our previous report [29]. Inhibition of Trpv4 with HC-067047 treatment at 0–24 hpt significantly reduced the numbers of pH3^+^ CMs (ventricle 0.6 ± 0.9, atrium 0.2 ± 0.4, N = 7). Inhibition of Notch signaling with DAPT treatment at 0–24 hpt produced a similar effect on CM proliferation reduction (ventricle 0.5 ± 0.7, atrium 0.5 ± 1.0, N = 7). However, the numbers of pH3^+^ CMs were only slightly reduced in the ventricle (7.0 ± 4.2, N = 8) and were not changed in the atrium (4.6 ± 2.5, N = 8) after the inhibition of NO synthesis with L-NMMA treatment at 0–24 hpt (Fig. 4F–I). The numbers of pH3^+^ CMs remained elevated at 72 hpt (ventricle 6.7 ± 2.4, atrium 2.8 ± 0.9, N = 13) (Fig. 4D’, E’, J), and HC-067047 treatment at 48–72 hpt exerted a strong inhibitory effect on CM proliferation (ventricle 1.3 ± 1.2, atrium 0.7 ± 0.9, N = 15). DAPT treatment at 48–72 hpt slightly reduced the number of pH3^+^ CMs in the ventricle (5.6 ± 2.0, N = 7) but had no effect in the atrium (2.6 ± 0.9, N = 7). Interestingly, L-NMMA treatment at 48–72 hpt markedly inhibited CM proliferation in the ventricle (3.4 ± 1.9, N = 15) but increased pH3^+^ CM numbers in the atrium (6.6 ± 2.3, N = 15) (Fig. 4F’-H’, J). Thus L-NMMA treatment during the later stage may have different effects on the proliferation of ventricular and atrial CMs, or it may affect the migration of proliferative CMs which were retained in the atrium.

Therefore, we used a previously reported cell lineage tracing system *Tg(amhc:CreERT2;β-actin2:loxP-DsRed-STOP-loxP-eGFP)* which could monitor the migratory behavior of genetical labelled CMs (cre-GFP^+^ CMs) during ventricle regeneration [4]. We examined the effects of inhibiting Trpv4, Notch and NO signaling at early and late stages on cre-GFP^+^ CM migration (Fig. 4K-Q). HC-067047 treatment at 0–24 or 48–72 hpt after ventricle ablation significantly reduced the contribution of cre-GFP^+^ CM in the ventricle compared to DMSO-treated ablated hearts (9.0% ± 4.3% vs 65.8% ± 4.8%, N = 7 for 0–24 hpt treatment, 9.8% ± 4.5% vs 63.4% ± 4.9%, N = 7 for 48–72 hpt treatment). DAPT and L-NMMA treatment exhibited opposite patterns. DAPT treatment at 0–24 hpt notably reduced the cre-GFP^+^ area in the ventricle (8.8% ± 3.1%, N = 7) but had less of an effect when treated at 48–72 hpt (57.4% ± 5.4%, N = 7). L-NMMA treatment at 0–24 hpt weakly reduced the cre-GFP^+^ area in the ventricle (57.2% ± 6.8%, N = 7) but exhibited strong inhibition when treated at 48–72 hpt (5.8% ± 3.1%, N = 7). These results suggested that the temporal requirements of Trpv4, Notch and NO signaling were different during ventricle regeneration. Trpv4 played an important role throughout the regeneration process, but Notch and NO signaling primarily functioned in the early and late stages of regeneration, respectively.

To further confirm the relationship of Trpv4, Notch and NO signaling and their temporal requirements, we used SNP to supply NO in ablated hearts in combination with HC-067047, DAPT or L-NMMA treatment at different time periods (Supplementary Fig. 7). NO replenishment by SNP for 48–72 hpt significantly increased the NO level and regeneration ratio (65%, N = 91 vs. 25%, N = 94) at 96 hpt, which was inhibited by HC-067047 treatment. However, HC-067047 combined with SNP treatment for 0–24 hpt did not influence the NO level or regeneration ratio (38%, N = 89 vs. 34%, N = 98) compared with HC-067047 treatment alone (Supplementary Fig. 7A, D). DAPT/SNP co-treatment had no effect on the NO level or regeneration ratio for 0–24 hpt (39%, N = 87 vs. 35%, N = 90) or 48–72 hpt (72%, N = 99 vs. 68%, N = 93) compared to DAPT treatment alone during the corresponding periods (Supplementary Fig. 7B, E). NO replenishment by SNP for 0–24 hpt slightly increased the regeneration ratio (72%, N = 81 vs. 61%, N = 88) at 96 hpt compared to the L-NMMA-treated ablated group, but SNP co-treatment for 48–72 hpt dramatically increased the NO level and regeneration ratio (68%, N = 95 vs. 35%, N = 89) that was inhibited by L-NMMA treatment (Supplementary Fig. 7C, F). We also used SNP to sustain production of NO in control and ablated hearts at 0–24 hpt. SNP treatment significantly increased the DAF-FM DA fluorescence in the BA of control and ablated hearts, but did not affect the activation of Notch signaling and regeneration ratio (Supplementary Fig. 8). Taken together, our results revealed that NO signaling functioned downstream of Trpv4 and majorly in the late stage of regeneration.

### Trpv4 and NO signaling play essential roles in the late stage of ventricle regeneration

**Fig. 5:**
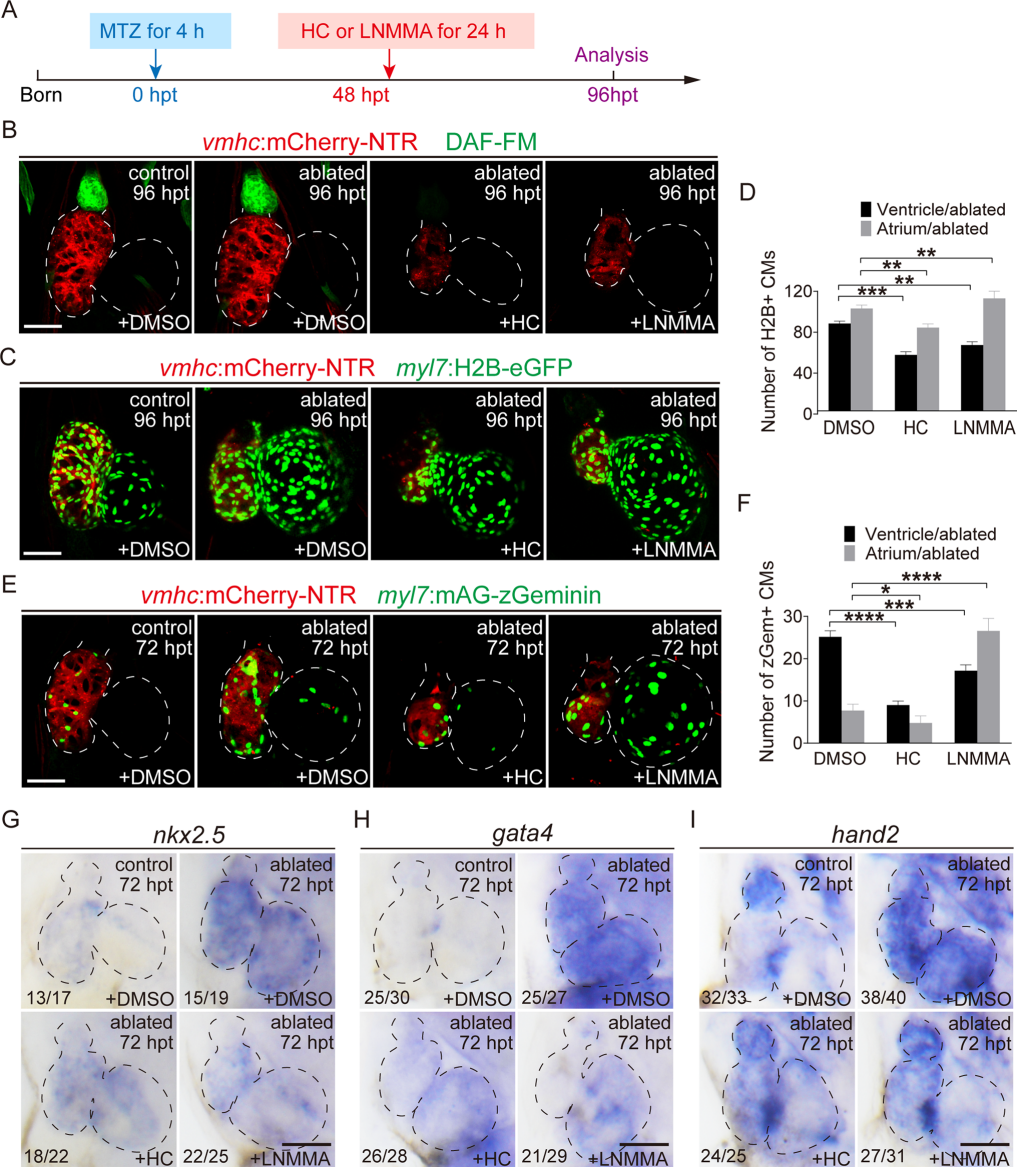
Trpv4 and NO signaling play essential roles in the late stage of ventricle regeneration. **A** Schematic timeline diagram of MTZ treatment to induce ventricle ablation and HC-067047 treatment to inhibit Trpv4 or L-NMMA treatment to inhibit NO synthesis. **B** DAF-FM DA staining of *Tg(vmhc:mCherry-NTR)* larvae at 96 hpt revealed that HC-067047 or L-NMMA treatment for 48–72 hpt blocked NO production. **C** Confocal images of control and ablated hearts treated with DMSO, HC-067047 or L-NMMA for 48–72 hpt in *Tg(vmhc:mCherry-NTR; myl7:H2B-eGFP)* larvae at 96 hpt. **D** Quantification of H2B^+^ CM numbers in DMSO-, HC-067047- and L-NMMA-treated ablated hearts. N = 19, 15, 10, respectively. Mean + s.e.m. ANOVA analysis; **P < 0.01, ***P < 0.001. **E** Confocal images of control and ablated hearts treated with DMSO, HC-067047 or L-NMMA for 48–72 hpt in *Tg(vmhc:mCherry-NTR; myl7:mAG-zGeminin)* larvae at 72 hpt. **F** Quantification of zGeminin^+^ CM numbers in DMSO-, HC-067047- and L-NMMA-treated ablated hearts. N = 17, 10, 15, respectively. Mean + s.e.m. ANOVA analysis; *P < 0.05, ***P < 0.001, ****P < 0.0001. **G**–**I** Whole-mount in situ hybridizations showed reduced expression of *nkx2.5*, *gata4* and *hand2* at 72 hpt in control and ablated hearts treated with DMSO, HC-067047 or L-NMMA for 48–72 hpt. Numbers indicate the ratio of representative staining observed. Scale bars, 50 μm. Dashed lines outline the hearts. *hpt* hours post treatment, *NO* nitric oxide, *CM* cardiomyocyte, *V* ventricle, *A* atrium, *HC* HC-067047

To further clarify the function of Trpv4 and NO in the late stage of ventricle regeneration, larvae were treated with HC-067047 or L-NMMA at 48 hpt for 24 h (Fig. 5A). DAF-FM DA staining showed that HC-067047 treatment significantly inhibited NO production in the BA and ventricle regeneration at 96 hpt in the ablated group, and the same effect was achieved following L-NMMA treatment at 48–72 hpt (Fig. 5B). We examined the change in CM numbers during ventricle regeneration using the *Tg(myl7:H2B-eGFP)* line, which specifically labels CM nuclei [20]. HC-067047 treatment at 48–72 hpt markedly decreased the number of ventricular CMs (49.0 ± 10.7, N = 15 vs. 76.3 ± 9.9, N = 17) and atrial CMs (72.8 ± 12.5, N = 15 vs. 89.5 ± 14.6, N = 17) in ablated hearts at 96 hpt compared to the DMSO-treated ablated group (Fig. 5C, D). L-NMMA treatment decreased the number of ventricular CMs (57.5 ± 8.8, N = 10) but increased the number of atrial CMs (98.3 ± 18.9, N = 10). These results were consistent with the pH3 staining results shown in Fig. 4J. HC-067047 or L-NMMA treatment did not affect the number of ventricular and atrial CMs in the non-ablated group (Supplementary Fig. 6C, D).

We next detected CM proliferation using transgenic line *Tg(vmhc:mCherry-NTR; myl7:mAG-zGeminin)*, which uses fluorescent ubiquitylation-based cell cycle indicator (FUCCI) technology [29]. Geminin expresses in the S/G2/M phases of cell cycle and is promptly degraded by the proteasome in the G1 phase, thus green fluorescence (mAG) indicates that these CMs are in the proliferating S/G2/M phases of cell cycle. HC-067047 treatment at 48–72 hpt dramatically inhibited the increase in zGem^+^ CMs in the ventricle (9.0 ± 2.9, N = 10 vs. 25.1 ± 6.1, N = 17) and atrium (4.7 ± 5.1, N = 10 vs. 7.7 ± 6.3, N = 17) in ablated hearts at 72 hpt compared to the DMSO-treated ablated group. However, L-NMMA treatment reduced the zGem^+^ CM number in the ventricle in a lesser degree (17.1 ± 5.6, N = 15) but significantly increased the zGem^+^ CM number in the atrium (26.5 ± 11.8, N = 15) in ablated hearts at 72 hpt (Fig. 5E, F). These results suggested that NO signaling affected CM proliferation in the ventricle and may affect the migration of proliferative CMs which were retained in the atrium. The re-expression of key early cardiac transcriptional regulators, such as *nkx2.5*, *gata4* and* hand2*, marks the de-differentiation of CMs and greatly contributes to CM proliferation and migration [4]. WISH showed that HC-067047 or L-NMMA treatment 48–72 h after ventricle ablation decreased the expression of *nkx2.5*, *gata4* and* hand2* compared to the DMSO-treated ablated group (Fig. 5G–I).

**Fig. 6:**
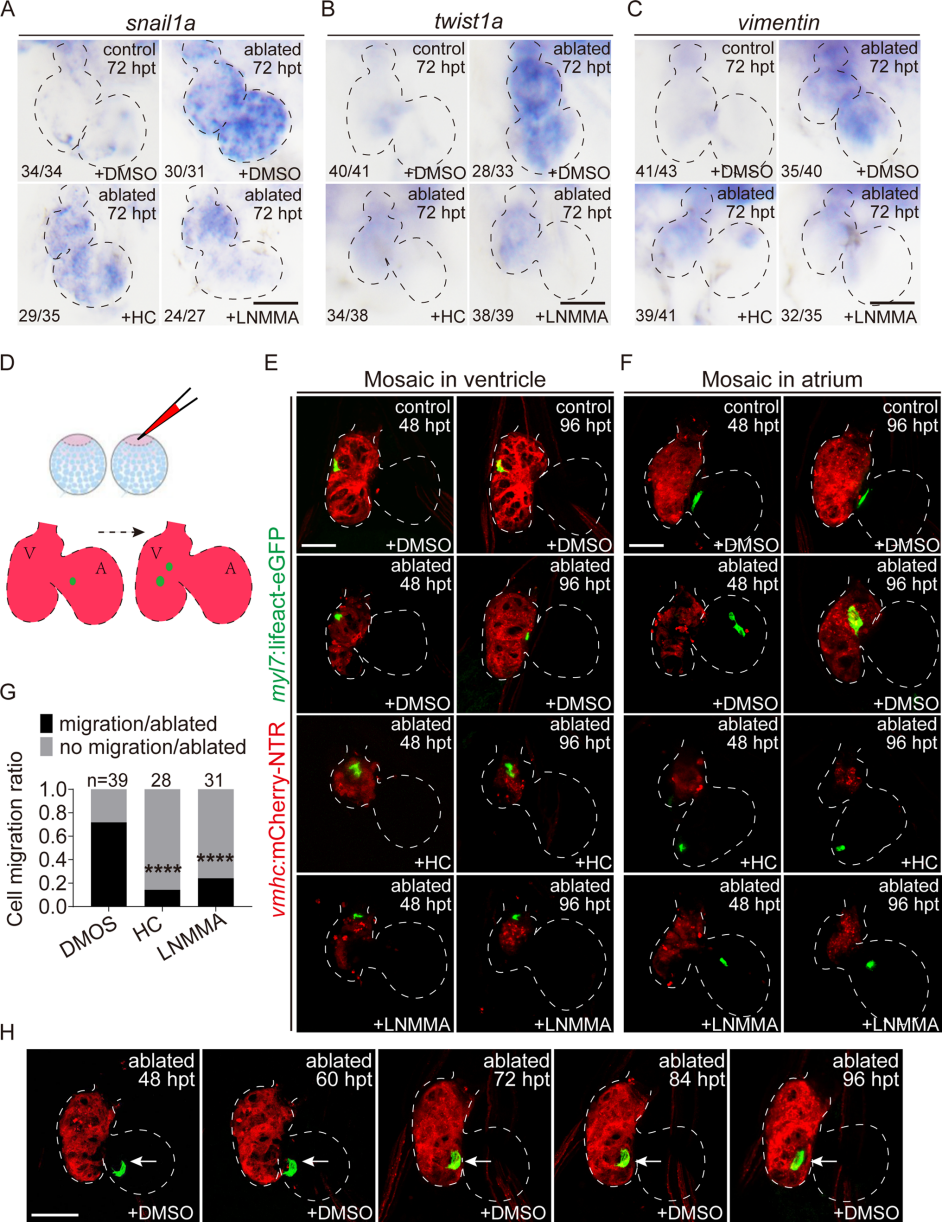
NO signaling regulates CM migration during ventricle regeneration. **A**–**C** Whole-mount in situ hybridizations showed reduced expression of *snail1a*, *twist1a* and *vimentin* at 72 hpt in control and ablated hearts treated with DMSO, HC-067047 or L-NMMA for 48–72 hpt. Numbers indicate the ratio of representative staining observed. **D**–**F** Injection of *myl7:*lifeact-eGFP plasmid into *Tg(vmhc:mCherry-NTR)* embryos at the one-cell stage to monitor the migratory behavior of single eGFP^+^ CMs in the ventricles or atriums of ablated hearts treated with DMSO, HC-067047 or L-NMMA for 48–72 hpt. **G** Quantification of the cell migration rate at 96 hpt in ablated hearts treated with DMSO, HC-067047 or L-NMMA for 48–72 hpt. The numbers of larvae analyzed for each condition are indicated. Binomial test, ****P < 0.0001. **H** Time series images showed the migration process of an eGFP^+^ CM in the ablated heart at 48–96 hpt. Scale bars, 50 μm. Dashed lines outline the hearts. *hpt* hours post treatment, *CM* cardiomyocyte, *V* ventricle, *A* atrium, *HC* HC-067047

We also examined the expression of several epithelial-mesenchymal transition (EMT) marker genes, such as *snail*, *twist*, and *vimentin*, which are important for CM migration [20]. WISH showed that HC-067047 or L-NMMA treatment 48–72 h after ventricle ablation decreased the expression of *snail1a*, *twist1a*, and *vimentin* compared to the DMSO-treated ablated group (Fig. 6A–C). To further examine the effects of drug treatment on CM migration, we injected the *myl7:*lifeact-eGFP plasmid into *Tg(vmhc:mCherry-NTR)* embryos at one-cell stage to obtain a mosaic expression of single CM cell with eGFP fluorescence and recorded its position between 48 and 96 h after ventricle ablation when the CMs were less proliferative (Fig. 6D). For the DMSO-treated ablated group, 72% of the single eGFP^+^ CMs (N = 28/39) exhibited migratory behavior within the ventricle or from the atrium to the ventricle. HC-067047 or L-NMMA treatment at 48–72 hpt greatly inhibited CM migration. Most single eGFP^+^ CMs remained static, and the migration ratio dropped to 14% (N = 4/28) and 22% (N = 7/31), respectively (Fig. 6E–H). These results were consistent with Fig. 4Q.

### NO signaling mediates ventricle regeneration through TGF-β pathway

**Fig. 7:**
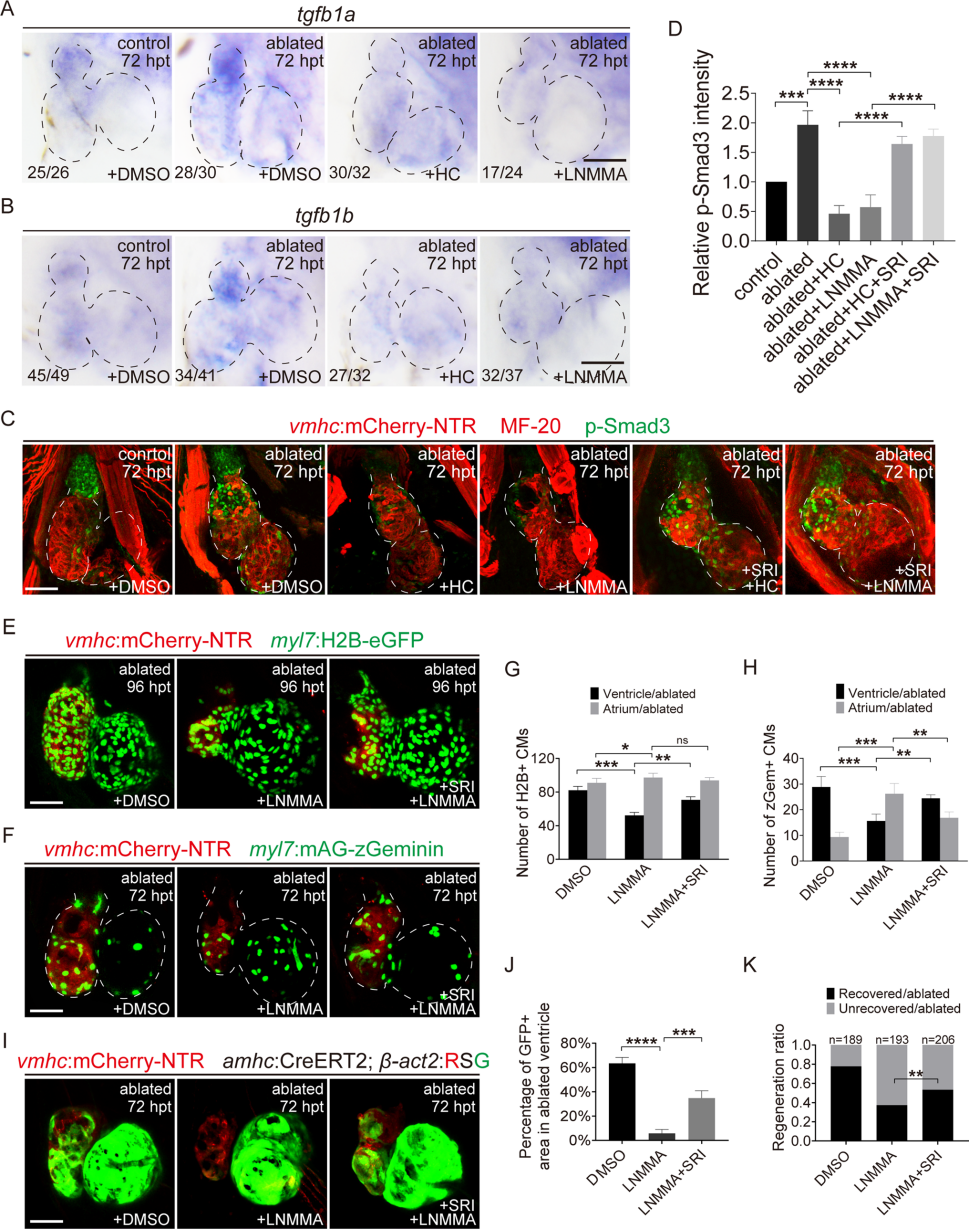
NO signaling mediates ventricle regeneration through TGF-β pathway. **A**, **B** Whole-mount in situ hybridizations showed the expression of *tgfb1a* and *tgfb1b* at 72 hpt in control and ablated hearts treated with DMSO, HC-067047 or L-NMMA for 48–72 hpt. Numbers indicate the ratio of representative staining observed. **C** Immunostaining of anti-myosin heavy chain (MF-20, red) and anti-phospho-Smad3 (green) in control and ablated *Tg(vmhc:mCherry-NTR)* hearts treated with DMSO, HC-067047 or L-NMMA alone or with SRI-011381 for 48–72 hpt. **D** Quantification of relative p-Smad3 intensity in control or ablated hearts with DMSO, HC-067047, L-NMMA or SRI-011381 treatment for 48–72 hpt. N = 7 for each group. Mean + s.e.m. ANOVA analysis; ***P < 0.001, ****P < 0.0001. **E**, **F** Confocal images of ablated hearts treated with DMSO, L-NMMA or L-NMMA + SRI-011381 for 48–72 hpt in *Tg(vmhc:mCherry-NTR; myl7:H2B-eGFP)* larvae at 96 hpt and in *Tg(vmhc:mCherry-NTR; myl7:mAG-zGeminin)* larvae at 72 hpt. **G**, **H** Quantification of H2B^+^ CM and zGeminin^+^ CM numbers in DMSO-, L-NMMA- and L-NMMA + SRI-011381-treated ablated hearts. N = 10 for each group. Mean + s.e.m. ANOVA analysis; ns, not significant; *P < 0.05, **P < 0.01, ***P < 0.001. **I** Confocal images of ablated hearts treated with DMSO, L-NMMA or L-NMMA + SRI-011381 for 48–72 hpt in *Tg(vmhc:mCherry-NTR; amhc:CreERT2; β-act2:RSG)* larvae at 72 hpt. **J** Quantification of the percentages of GFP-positive area in DMSO-, L-NMMA- and L-NMMA + SRI-011381-treated ablated hearts. N = 10 for each group. Mean + s.e.m. ANOVA analysis; ***P < 0.001, ****P < 0.0001. **K** Quantification of the heart recovery rate in ablated groups treated with DMSO, L-NMMA, L-NMMA + SRI-011381 at 96 hpt. The numbers of larvae analyzed for each condition are indicated. Binomial test, **P < 0.01. Scale bars, 50 μm. Dashed lines outline the hearts. *hpt* hours post treatment, *CM* cardiomyocyte, *HC* HC-067047, *SRI* SRI-011381

Subsequently, we explored how NO signal in the BA regulates the cellular processes of CMs. TGF-β are secretory growth factors which regulated CM proliferation and migration in ablated hearts as shown in our previous study [20]. WISH showed that TGF-β ligand *tgfb1a* mainly expressed in the BA of control hearts. Its expression was dramatically increased in ablated hearts at 72 hpt and abolished by HC-067047 or L-NMMA treatment for 48–72 hpt (Fig. 7A). The upregulation of another TGF-β ligand *tgfb1b* in ablated hearts was also inhibited by HC-067047 or L-NMMA treatment (Fig. 7B). Immunofluorescence staining revealed that high level of phospho-Smad3 (p-Smad3), the active form of TGF-β/Smad signaling pathway, was mainly restricted in the BA of control hearts and elevated in the whole hearts 72 h after ablation, both in the myocardium and epicardium. Treatment with HC-067047 or L-NMMA at 48–72 hpt significantly reduced p-Smad3 level in ablated hearts (Fig. 7C, D, Supplementary Fig. 9). Co-treatment of SRI-011381, a TGF-β signaling agonist [30], activated TGF-β pathway and increased p-Smad3 level (Fig. 7C, D). More importantly, co-treatment of SRI-011381 ameliorated the inhibitory effects of NO synthesis blockage on CM proliferation (Fig. 7E–H) and migration (Fig. 7I, J). The heart recovery ratio at 96 hpt increased to 63% in the ablated group co-treated with L-NMMA and SRI-011381 (N = 206), compared to the 46% recovery rate in the ablated group treated with L-NMMA alone (N = 193) (Fig. 7K). These results suggested that NO mediates CM proliferation and migration by regulating TGF-β/Smad signaling pathway during ventricle regeneration.

## Discussion

The present study revealed a signaling cascade by which the heart transmits hemodynamic changes after injury to promote the de-differentiation, proliferation, and migration of CMs for cardiac repair and regeneration. After ventricle ablation, hemodynamic alteration is perceived by the mechanosensitive channel Trpv4, which not only regulates *klf2a* expression and Notch signaling in the AVC, as reported in our previous study [4, 7], but also modulates NO signaling in the BA to mediate cardiac regeneration. Although both Notch and NO signaling pathways are modulated by blood flow and Trpv4, they function in heart regeneration at different stages and locations, cooperatively regulating multiple cellular events (Supplementary Fig. 10).

### The role of NO in heart regeneration

With strong lipophilicity, NO easily penetrates the cell membrane and functions as an important signal transduction molecule in cardiovascular development and diseases [22]. NO in the mammalian heart is primarily produced in myocardial cells, endocardial cells, intracardiac arteries, veins and the capillary network [31]. DAF-FM DA staining in our study showed that the NO signal was present in the smooth muscle layer of BA in zebrafish, which is consistent with previous studies from other groups [24, 32]. Although NO plays important roles in hematopoiesis, vasodilation, platelet aggregation and myocardial contraction [14–16], the role of NO in the regulation of cardiac regeneration has not been well studied. Ma et al. revealed that the addition of exogenous NO in the early stage of ischemia reperfusion significantly reduced the necrotic area and prevented the occurrence of cardiogenic shock after reperfusion [33]. Rochon et al. discovered that nitrite treatment at physiological levels restored the ventricular area of zebrafish hearts after ventricular resection or cryoinjury in hypoxic water [34]. However, there is no evidence that NO directly influences cardiac regeneration so far. Our present study discovered dynamic changes in NO levels in the BA after ventricle ablation that were regulated by blood flow and Trpv4. Notably, we showed that the inhibition of NO production by NO synthase inhibitor dramatically impeded ventricle regeneration and supplementation of exogenous NO using an NO donor relieved the inhibition of regeneration. Our results provide direct evidence for the participation of NO in cardiac regeneration.

How NO signal in the BA affects ventricle regeneration is not clear and we speculated that NO may exert this function by establishing gradients of certain signaling molecules. The TGF-β signaling pathway regulates the differentiation, proliferation, and migration of a variety of cell types during cardiovascular development [35] and is also vital for heart regeneration [36]. The three TGF-β ligands are secreted in latent forms and disulfide-linked to one of three latent TGF-β binding proteins (LTBPs 1, 3, and 4), forming a large latent complex (LLC) which anchors to the extracellular matrix prior to ligand activation. Activated by integrins, proteases, or matrix proteins, latent TGF-β proteins are released from the complex, bind to the TGFBR2 and TGFBR1 receptors on cell membranes, and then activate downstream Smad-dependent or Smad-independent signaling pathways [37, 38]. Previous study showed that *ltbp3* gene was specifically expressed in the zebrafish BA at 2–5 dpf [30]. Zhou et al*.* confirmed that *ltbp3*^+^ cells gave rise to distal ventricular myocardium through late differentiation and accretion to the heart tube. They also demonstrated that pSmad2 signal was observed in the heart tube and in extra-cardiac cells. Loss of *ltbp3* function eliminated pSmad2 epitopes specifically in the extra-cardiac population [39]. Our recent study showed that TGF-β/Smad signaling pathway was involved in CM proliferation and migration in ablated zebrafish hearts [20]. In present study, TGF-β ligand *tgfb1a* and p-Smad3 signals were mainly present in the BA of control hearts and strongly increased in the ablated hearts, which were abolished by treatment of Trpv4 antagonist HC-067047 or NO synthase inhibitor L-NMMA. Co-treatment of a TGF-β signaling agonist SRI-011381 ameliorated the inhibitory effects of NO synthesis blockage on CM proliferation, migration and ventricle regeneration. Our results also revealed that TGF-β signaling was mainly activated in the myocardium and epicardium in ablated hearts. This finding was similar to a recent report that Hedgehog signaling in the cardiac outflow tract was sufficient to direct regeneration of the adjacent ventricular epicardium [40]. Taken together, we hypothesized that NO may affect the secretion of TGF-β from the BA to the myocardium directly or through the epicardium, thereby mediating CM proliferation and migration in ablated hearts, but the mechanism warrants further investigation.

### Trpv4 regulates ventricle regeneration through spatiotemporal modulation of Notch and NO signaling

TRPV4 is a mechanosensitive ion channel located on the cell membrane [12]. Our previous report showed that the injured heart sensed altered intracardiac hemodynamic forces, and Trpv4 regulated the endocardial Klf2a-Notch signaling cascade to modulate the myocardial BMP and Erbb2 signaling pathways and promote cardiac reprogramming and repair [6, 7]. The present study further revealed that Trpv4 was ubiquitously expressed in myocardial and endocardial cells including the AVC and BA. Trpv4 expression was significantly upregulated after ventricle ablation and dramatically blocked by reduced blood flow. The addition of Trpv4 agonist 4α-pdd partially rescued Notch activation that was suppressed by tricaine in ablated hearts.

TRPV4 has also been implicated in NO signal regulation. During regeneration of the arterial circulation, TRPV4 activation transforms blood flow shear forces into cell growth signals, stimulates the release of NO and causes the proliferation of vascular smooth muscle cells, which induces the growth of collateral vessels [41]. Mendoza et al. showed that NO and endothelium-derived hyperpolarizing factor (EDHF) components of flow-mediated relaxation were markedly reduced in *Trpv4*^−/−^ mice [42]. Activation of TRPV4 channels with GSK1016790A increased NO level in endothelial cell (EC) and smooth muscle cell (SMC) layers in the small pulmonary arteries (PAs) of wild-type, but not in *eNOS*^−/−^ mice [13]. Therefore, we speculate that Trpv4 also mediates zebrafish ventricle regeneration by regulating NO. Our results showed that treatment with Trpv4 agonist 4α-pdd partially restored NO levels that were suppressed by reduced blood flow, and *trpv4* deficiency by gene knockout or antagonist treatment significantly inhibited NO production and cardiac recovery during zebrafish ventricle regeneration. Although endocardial cells are the major responders for altered shear forces, we cannot rule out the possibility that Trpv4 may have functions in other cardiac cell types and need to perform tissue-specific analysis in the future.

Although both were modulated by Trpv4, Notch and NO signaling regulated zebrafish heart regeneration in a spatiotemporally different manner. Multiple overlapped cellular events, namely de-differentiation, proliferation, and migration of CMs, were orchestrated to ensure the success of heart regeneration [4]. The critical influence of endocardial Notch signaling around the AVC occurred in the early stage of regeneration, and the main effect of NO signaling in the BA occurred in the late stage of regeneration. They functioned cooperatively to regulate these cellular events. This temporal and spatial regulation by multiple signal pathways has been widely observed in many settings. Munch et al. revealed a highly dynamic endocardium after cryoinjury and identified *serpine1* as an early endocardial injury-response gene followed by Notch signaling activation during regeneration [43]. Spatial and temporal variations in shear stress differentially modulate critical steps in heart development, such as trabeculation and compaction of the ventricular wall and the formation of heart valves [44]. Mercer et al. also described that a dynamic spatiotemporal extracellular matrix facilitated epicardial-mediated vertebrate heart regeneration [45].

In summary, the present study demonstrated temporal and spatial requirements for NO and Notch signaling and their coordination during zebrafish ventricle regeneration. These findings reveal the critical role of the mechanosensitive channel Trpv4 in regulating heart regeneration and provide novel insights and new directions for the treatment of ischemic heart diseases.

### Supplementary Information

Below is the link to the electronic supplementary material.

Supplementary file1 (PDF 5265 KB)

## Data Availability

All data supporting the findings of this study are available within the article and the Supplementary Materials.
