# Supplementary material for: Spatiotemporal modulation of nitric oxide and Notch signaling by hemodynamic-responsive Trpv4 is essential for ventricle regeneration
Source: Cell Mol Life Sci. 2024 Jan 27;81(1):60. doi: 10.1007/s00018-023-05092-0 (PMC10817848; doi:10.1007/s00018-023-05092-0)
Supplement: Supplementary file 1 — Supplementary file1 (PDF 5265 KB) [file 18_2023_5092_MOESM1_ESM.pdf]

Supplementary materials

Supplementary Figure 1. DAF-FM DA staining of the hearts and whole larvae under various conditions

Supplementary Figure 2. NO production is reduced in *nos1* and *nos2b* crispants

Supplementary Figure 3. Generation and characterization of *trpv4*$^{-/-}$ mutants

Supplementary Figure 4. *trpv4* deficiency results in reduced cardiomyocyte proliferation and failure of ventricle regeneration

Supplementary Figure 5. Trpv4 modulates Notch signaling in the early stage of ventricle regeneration

Supplementary Figure 6. Treatment with inhibitors does not affect zebrafish heart development

Supplementary Figure 7. NO signaling acts downstream of Trpv4 and majorly in the late stage of regeneration

Supplementary Figure 8. NO supplement in the early stage of regeneration does not affect Notch signaling activation and heart regeneration

Supplementary Figure 9. TGF-β signaling is mainly activated in the epicardium and myocardium in ablated hearts

Supplementary Figure 10. Schematic diagram of spatiotemporal modulation of NO and Notch signaling by hemodynamic-responsive Trpv4 during ventricle regeneration

Supplementary Table 1. List of primers used in this study

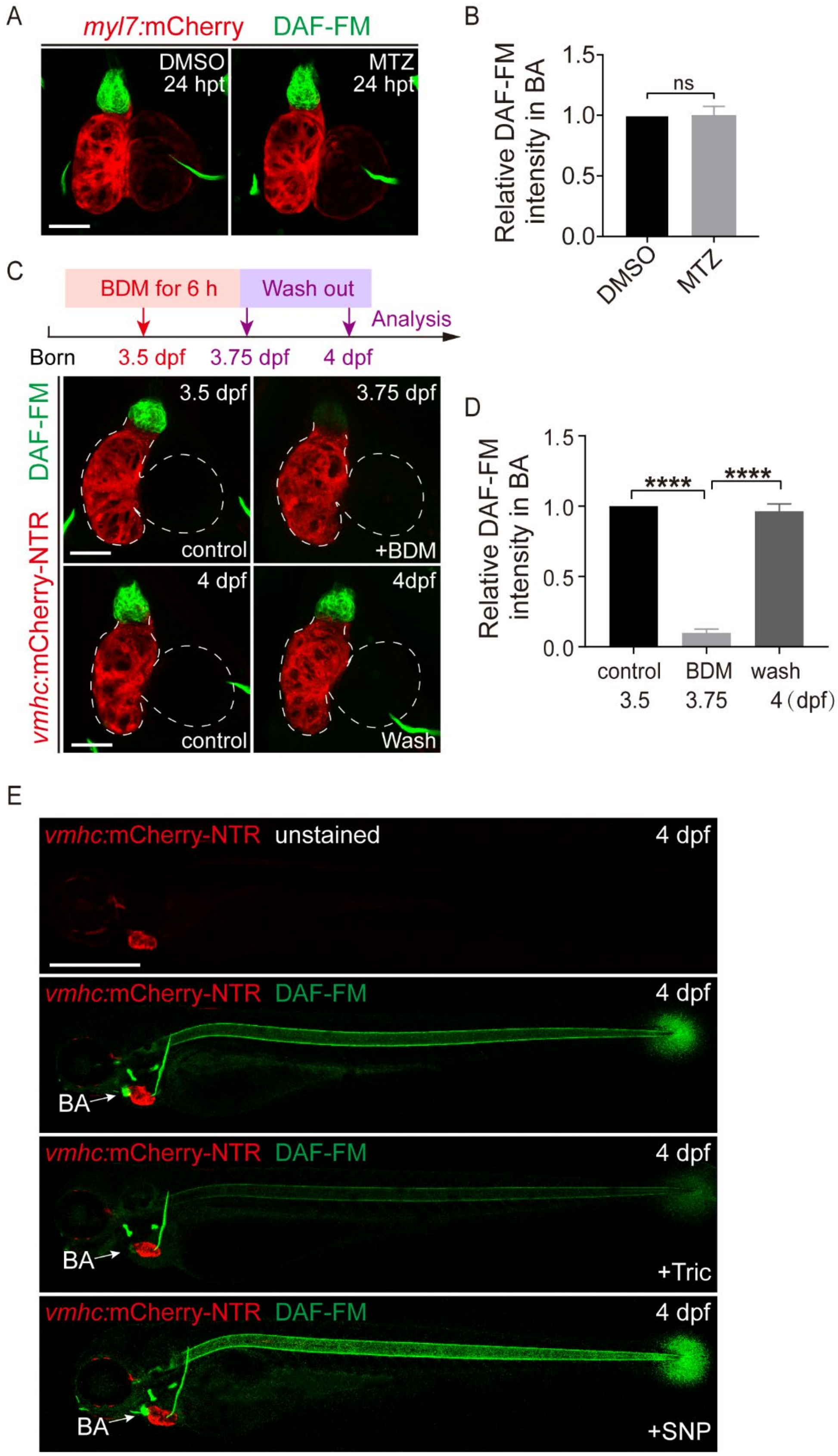

Supplementary Figure 1. DAF-FM DA staining of the hearts and whole larvae under various conditions

(A) DAF-FM DA staining of *Tg(myl7:mCherry)* hearts with DMSO or MTZ treatment at 24 hpt. hpt, hours post treatment; MTZ, metronidazole.

(B) Quantification of relative DAF-FM DA intensity of BA in DMSO or MTZ treated hearts at 24 hpt. N = 5 for each group. Mean + s.e.m. Student's t-test; ns, not significant.

(C) DAF-FM DA staining of *Tg(vmhc:mCherry-NTR)* hearts with BDM treatment at 3.5-4 dpf. dpf, days post fertilization; BDM, 2,3-butanedione monoxime.

(D) Quantification of relative DAF-FM DA intensity of BA in control, BDM treated or wash-out groups. N = 5 for each group. Mean + s.e.m. ANOVA analysis, ****$P < 0.0001$.

(E) DAF-FM DA staining of *Tg(vmhc:mCherry-NTR)* larvae with tricaine (Tric) or SNP treatment at 4 dpf. White arrows point to the bulbus arteriosus (BA).

Scale bars, (A, C) 50 μm, (E) 150 μm. Dashed lines outline the heart. dpf, days post fertilization; SNP, sodium nitroprusside dihydrate.

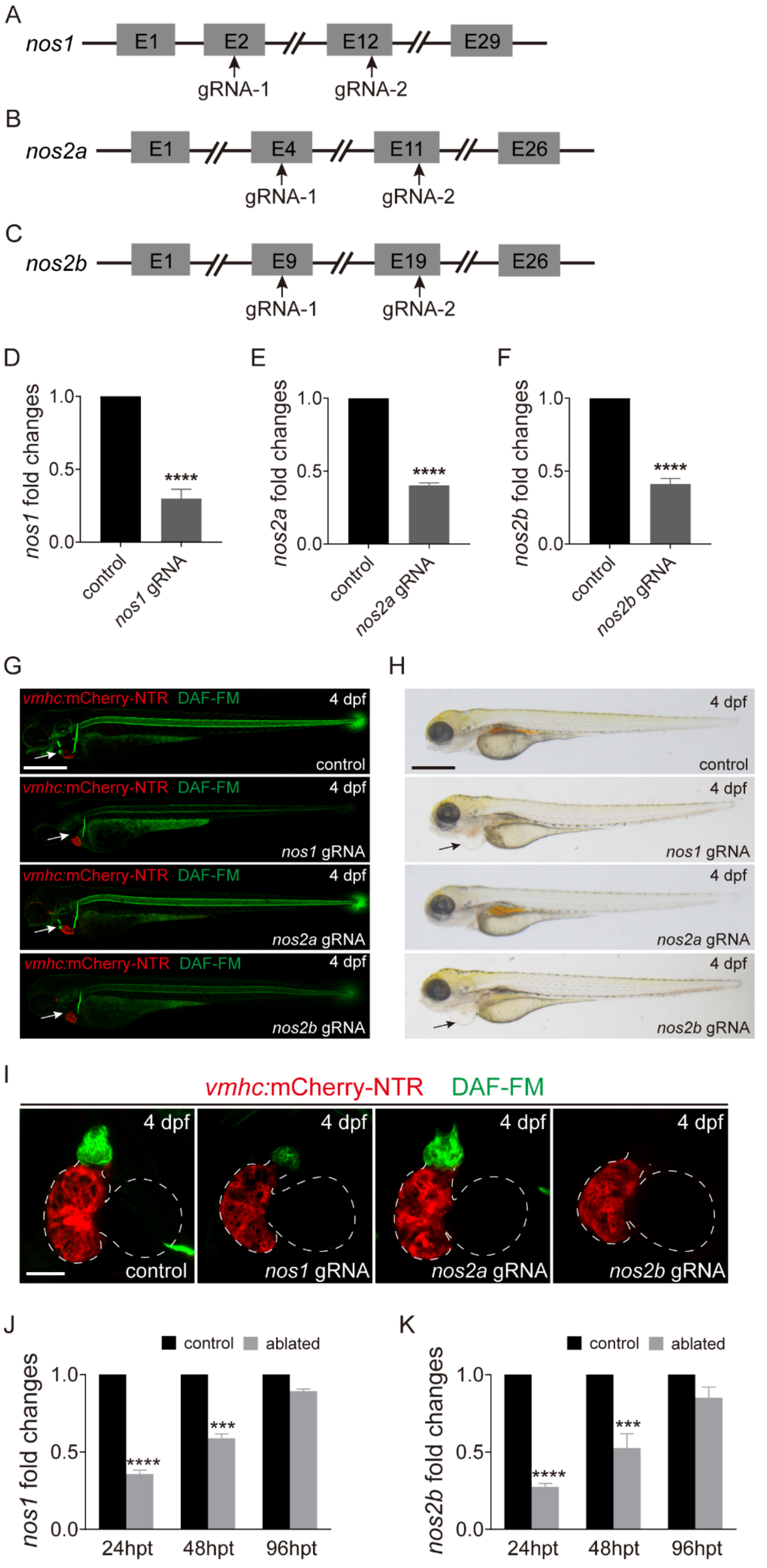

A
nos1
E1
E2
E12
E29
gRNA-1
gRNA-2
B
nos2a
E1
E4
E11
E26
gRNA-1
gRNA-2
C
nos2b
E1
E9
E19
E26
gRNA-1
gRNA-2
D
nos1 fold changes
1.0
0.5
0.0
****
control
nos1 gRNA
E
nos2a fold changes
1.0
0.5
0.0
****
control
nos2a gRNA
F
nos2b fold changes
1.0
0.5
0.0
****
control
nos2b gRNA
G
vmhc:mCherry-NTR DAF-FM
4 dpf
control
nos1 gRNA
nos2a gRNA
nos2b gRNA
H
4 dpf
control
nos1 gRNA
nos2a gRNA
nos2b gRNA
I
vmhc:mCherry-NTR DAF-FM
4 dpf
control
nos1 gRNA
nos2a gRNA
nos2b gRNA
J
control
ablated
nos1 fold changes
1.0
0.5
0.0
****
***
24hpt
48hpt
96hpt
K
control
ablated
nos2b fold changes
1.0
0.5
0.0
****
***
24hpt
48hpt
96hpt

Supplementary Figure 2. NO production is reduced in *nos1* and *nos2b* crispants

(A-C) Schematic diagrams of the zebrafish *nos1*, *nos2a* and *nos2b* loci with sgRNA target sites.

(D-F) Quantification of the fold change of *nos1*, *nos2a* and *nos2b* expression between control and crispant larvae at 4 dpf by real-time PCR. 3 independent experiments. Mean + s.e.m. Student's t-test, ****$P < 0.0001$.

(G) DAF-FM DA staining of *Tg(vmhc:mCherry-NTR)* larvae in control and crispant groups at 4 dpf. White arrows point to the bulbus arteriosus (BA).

(H) Morphology of control and crispant larvae at 4 dpf. Black arrows point to the pericardiac edema.

(I) DAF-FM DA staining of *Tg(vmhc:mCherry-NTR)* hearts in control and crispant groups at 4 dpf, the *nos1* and *nos2b* crispants exhibited abnormal heart development.

(J, K) Quantification of the fold change of *nos1* and *nos2b* expression between control and ablated hearts at 24, 48 and 96 hpt by real-time PCR. 3 independent experiments. Mean + s.e.m. Student's t-test, ***$P < 0.001$.

Scale bars, (G, H) 150 μm, (I) 50 μm. Dashed lines outline the heart. dpf, days post fertilization; hpt, hours post treatment.

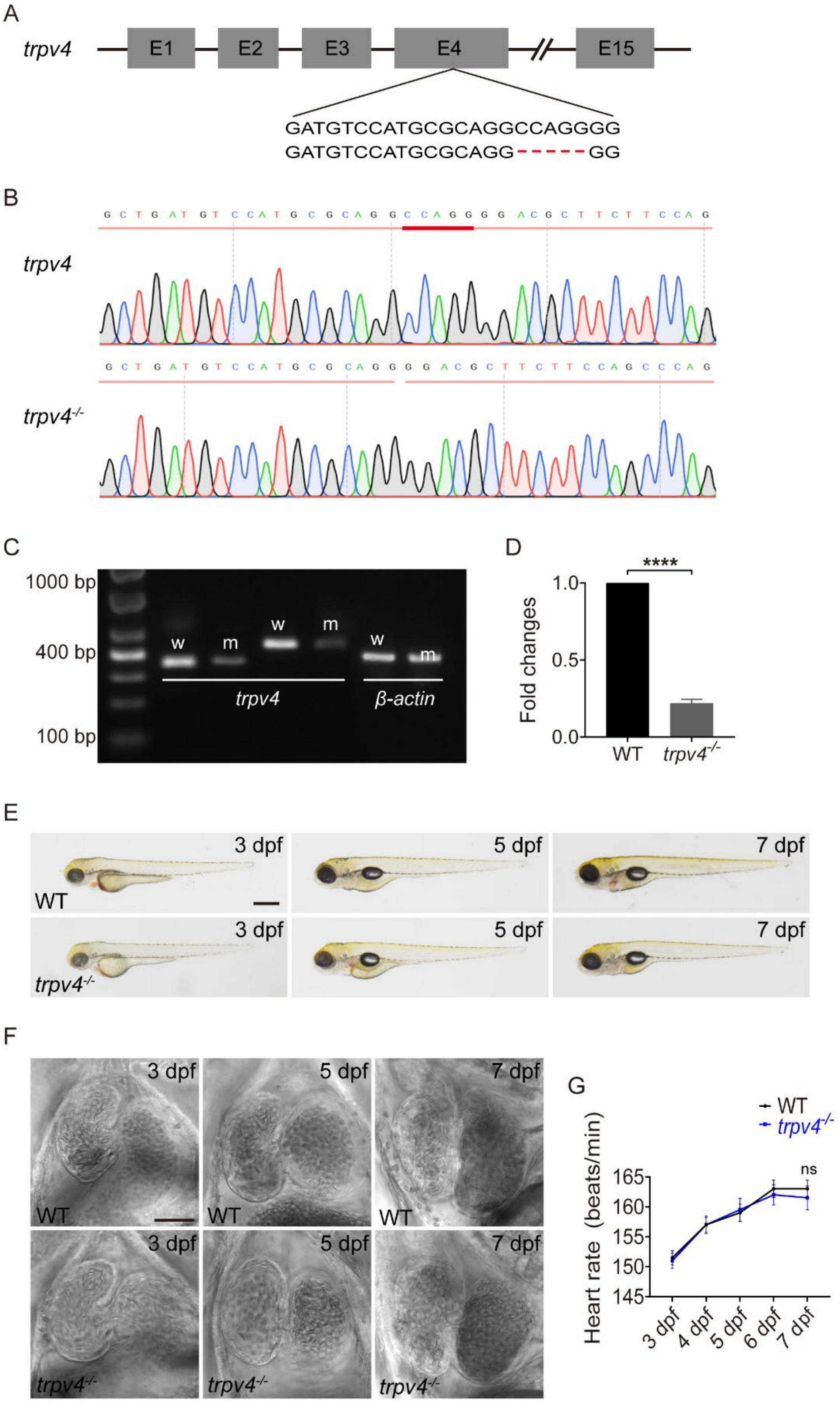

Supplementary Figure 3. Generation and characterization of *trpv4*$^{-/-}$ mutants

(A) Schematic diagram of the zebrafish *trpv4* locus with sgRNA target site sequence.

(B) A 5-bp deletion in *trpv4*$^{-/-}$mutants was confirmed by sequencing.

(C) Semi-quantitative RT-PCR analysis showed reduced *trpv4* transcript levels between wild-type (w) and mutant (m) larvae at 4 dpf. Two pairs of primers were used. β-actin was used as a control.

(D) Quantification of the fold change of *trpv4* expression between wild-type and mutant larvae at 4 dpf by real-time PCR. 4 independent experiments. Mean + s.e.m. Student's t-test, ****$P < 0.0001$.

(E) Morphology of wild-type and *trpv4* mutant larvae at 3, 5 and 7 dpf. Scale bar, 150 μm. dpf, days post fertilization.

(F) Heart morphology of wild-type and *trpv4* mutant larvae at 3, 5 and 7 dpf. Scale bar, 50 μm.

(G) Quantification of the heart rates in wild-type and *trpv4*$^{-/-}$mutant larvae from 3 to 7 dpf. N = 7 for each group. Mean + s.e.m. Student's t-test; ns, not significant.

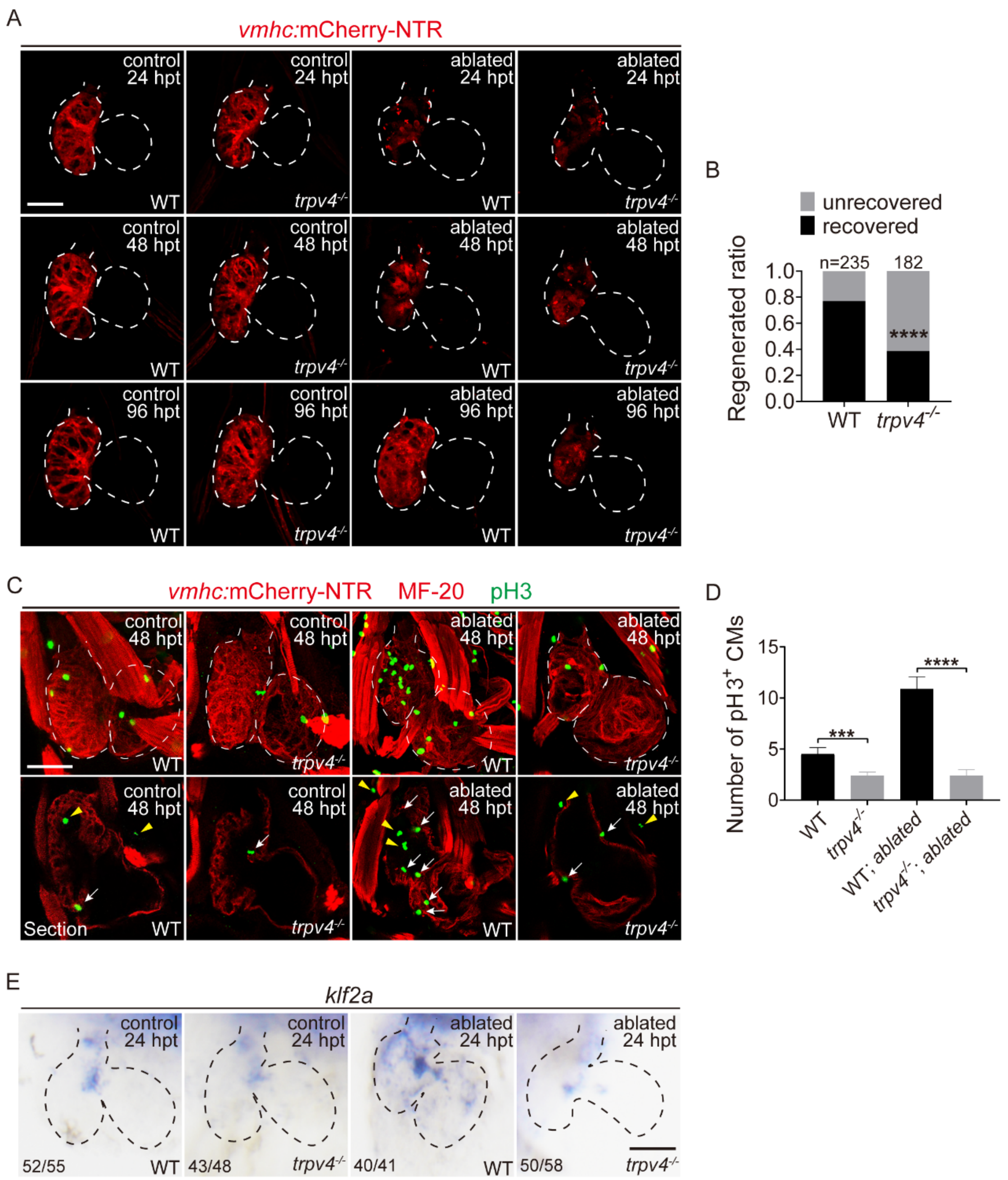

Supplementary Figure 4. *trpv4* deficiency results in reduced cardiomyocyte proliferation and failure of ventricle regeneration

(A) Confocal images of *Tg(vmhc:mCherry-NTR)* showed that ventricle regeneration was significantly inhibited in *trpv4*$^{-/-}$ mutants.

(B) Quantification of the heart recovery rate in ablated wild types and *trpv4*$^{-/-}$ mutants. The numbers of larvae analyzed for each condition are indicated. Binomial test; ****$P < 0.0001$.

(C) Immunostaining of anti-phospho-histone H3 (pH3, green) and anti-myosin heavy

chain (MF-20, red) revealed that the numbers of proliferating cardiomyocytes were dramatically reduced in *trpv4*$^{-/-}$ mutants compared with wild types after ablation at 48 hpt. Section images revealed proliferating cardiomyocytes (white arrows) and proliferating non-cardiomyocytes (yellow arrowheads).

(D) Quantification of pH3^{+} cardiomyocyte numbers in wild types and *trpv4*$^{-/-}$ mutants at 48 hpt. N = 10 for each group. Mean + s.e.m. ANOVA analysis, ***$P < 0.001$, ****$P < 0.0001$.

(E) Whole-mount *in situ* hybridizations of *klf2a* expression in wild types and *trpv4*$^{-/-}$ mutants at 24 hpt.

Scale bars, 50 μm. Dashed lines outline the hearts. hpt, hours post treatment.

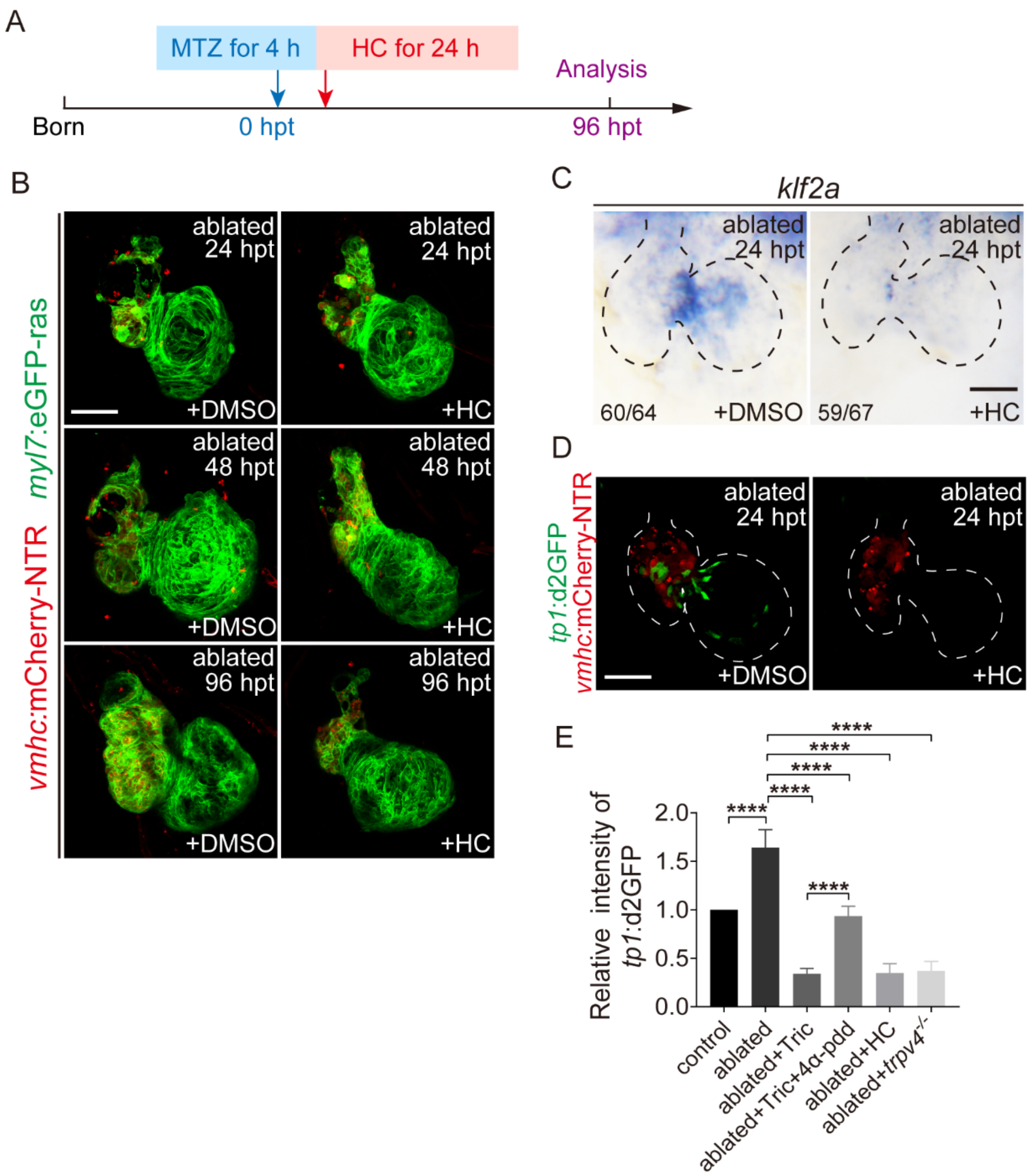

Supplementary Figure 5. Trpv4 modulates Notch signaling in the early stage of ventricle regeneration

(A) Schematic timeline diagram of MTZ treatment to induce ventricle ablation and HC-067047 treatment to inhibit Trpv4.

(B) Confocal images of *Tg(vmhc:mCherry-NTR; myl7:eGFP-ras)* hearts showed that HC-067047 treatment for 0-24 hpt significantly inhibited ventricle regeneration.

(C) Whole-mount *in situ* hybridizations indicated that *klf2*a upregulation in ablated hearts at 24 hpt was blocked by HC-067047 treatment for 0-24 hpt. Numbers indicate the ratio of representative staining observed.

(D) Confocal images of *Tg(vmhc:mCherry-NTR; tp1:d2GFP)* hearts showed that HC-067047 treatment for 0-24 hpt markedly abolished Notch signaling activation in ablated hearts at 24 hpt.

(E) Quantification of relative *tp1*:d2GFP intensity in control hearts, ablated hearts treated with DMSO, Tricaine, Tricaine + 4α-pdd or HC-067047 for 0-24 hpt, and ablated *trpv4*$^{-/-}$ mutant hearts. N = 7 for each group. Mean + s.e.m. ANOVA analysis; ****P < 0.0001.

Scale bars, 50 μm. Dashed lines outline the hearts. hpt, hours post treatment; HC, HC-067047; Tric, Tricaine.

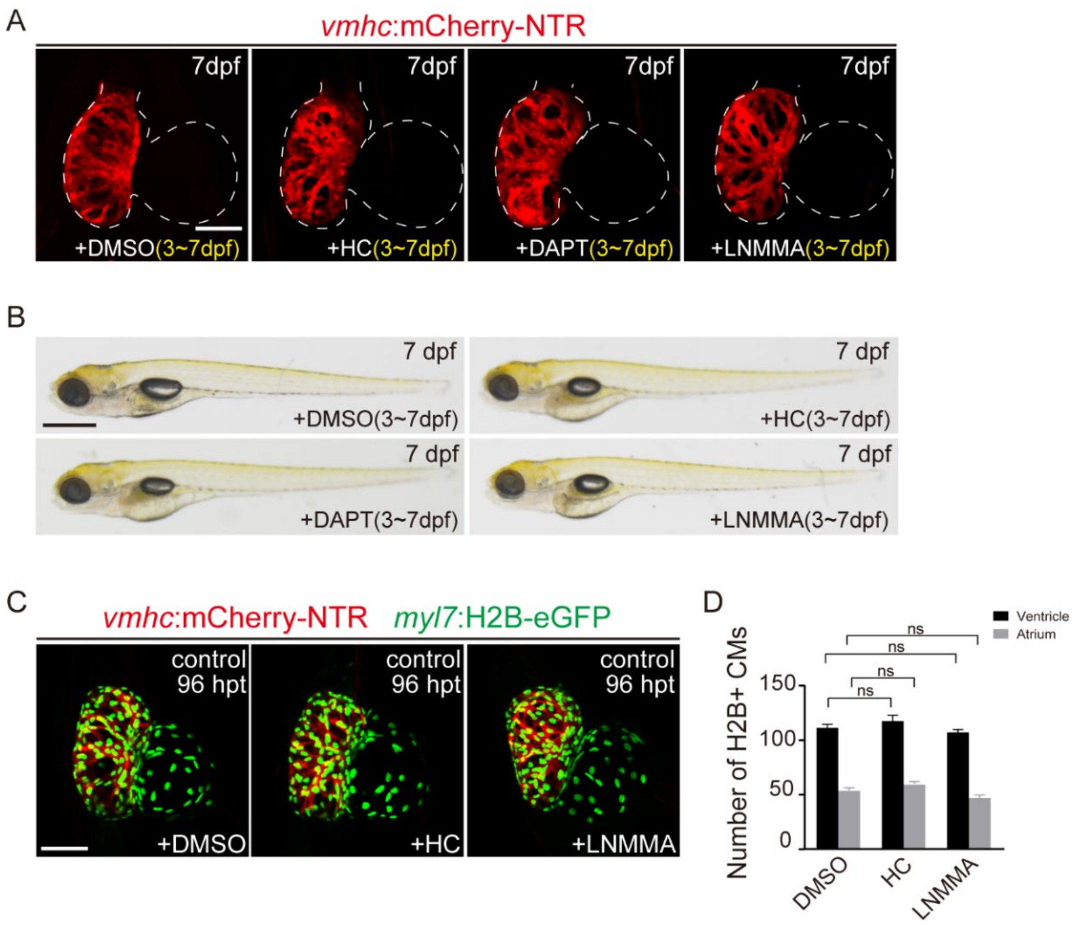

Supplementary Figure 6. Treatment with inhibitors does not affect zebrafish heart development

(A) Confocal images of hearts treated with DMSO, HC-067047, DAPT or L-NMMA for 3-7 dpf in *Tg(vmhc:mCherry-NTR)* larvae at 7 dpf. Scale bar, 50 µm.

(B) Morphology of larvae at 7 dpf treated with DMSO, HC-067047, DAPT or L-NMMA for 3-7 dpf. Scale bar, 150 µm.

(C) Confocal images of control hearts treated with DMSO, HC-067047 or L-NMMA for 48-72 hpt in *Tg(vmhc:mCherry-NTR; myl7:H2B-eGFP)* larvae at 96 hpt. Scale bar, 50 µm.

(D) Quantification of $H2B^+$ CM numbers in DMSO-, HC-067047- and L-NMMA-treated non-ablated hearts. N = 15, 12, 10, respectively. Mean + s.e.m. ANOVA analysis; ns, not significant.

Dashed lines outline the hearts. hpt, hours post treatment; dpf, days post fertilization; HC, HC-067047.

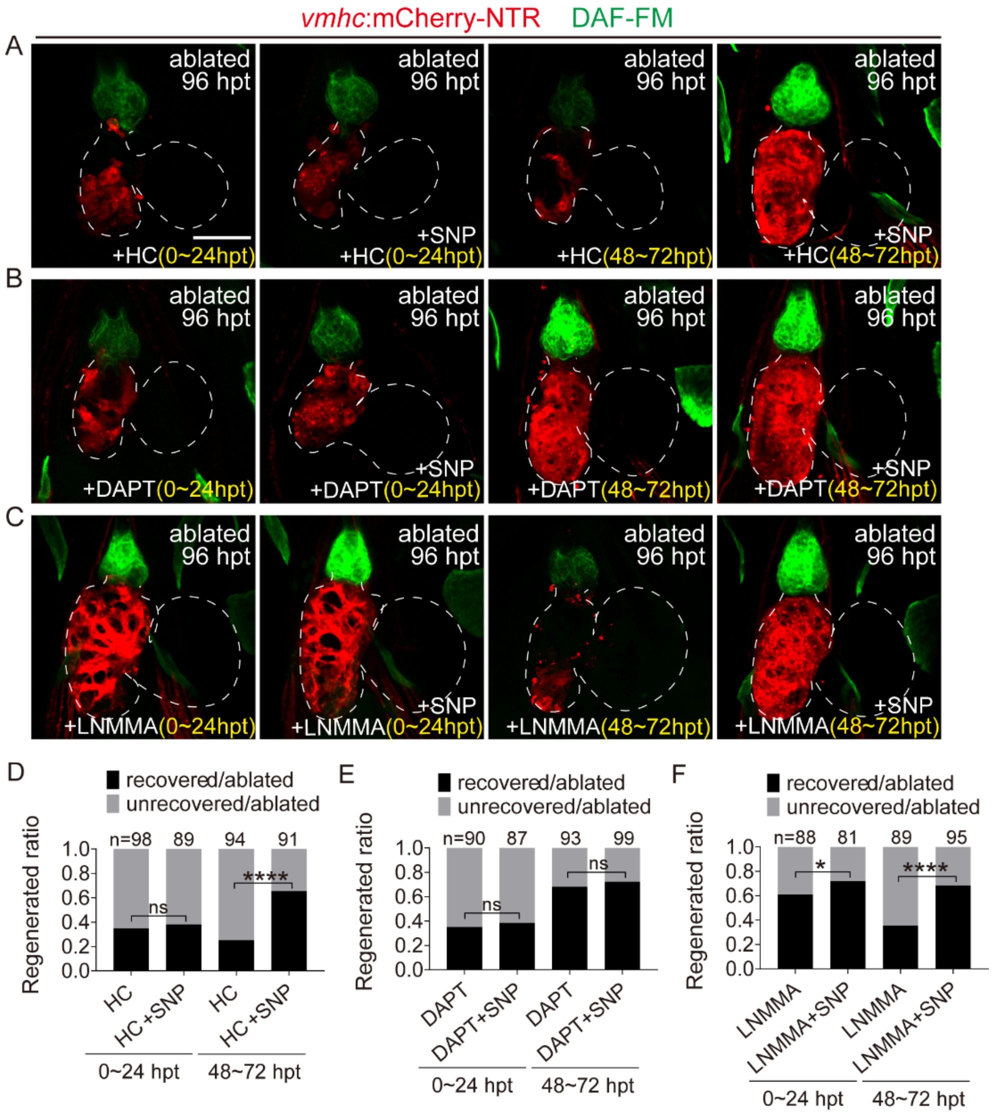

Supplementary Figure 7. NO signaling acts downstream of Trpv4 and majorly in the late stage of regeneration

(A-C) DAF-FM DA staining of *Tg(vmhc:mCherry-NTR)* ablated hearts at 96 hpt treated with HC-067047 (A), DAPT (B) or L-NMMA (C) for 0-24 or 48-72 hpt, with or without supplement of NO donor SNP.

(D-F) Quantification of the heart recovery rate in ablated groups at 96 hpt treated with HC-067047 (D), DAPT (E) or L-NMMA (F) for 0-24 or 48-72 hpt, with or without supplement of NO donor SNP. The numbers of larvae analyzed for each condition are indicated. Binomial test; ns, not significant; *$P < 0.05$, ****$P < 0.0001$.

Scale bars, 50 μm. Dashed lines outline the hearts. hpt, hours post treatment; NO, nitric oxide; SNP, sodium nitroprusside dihydrate; HC, HC-067047.

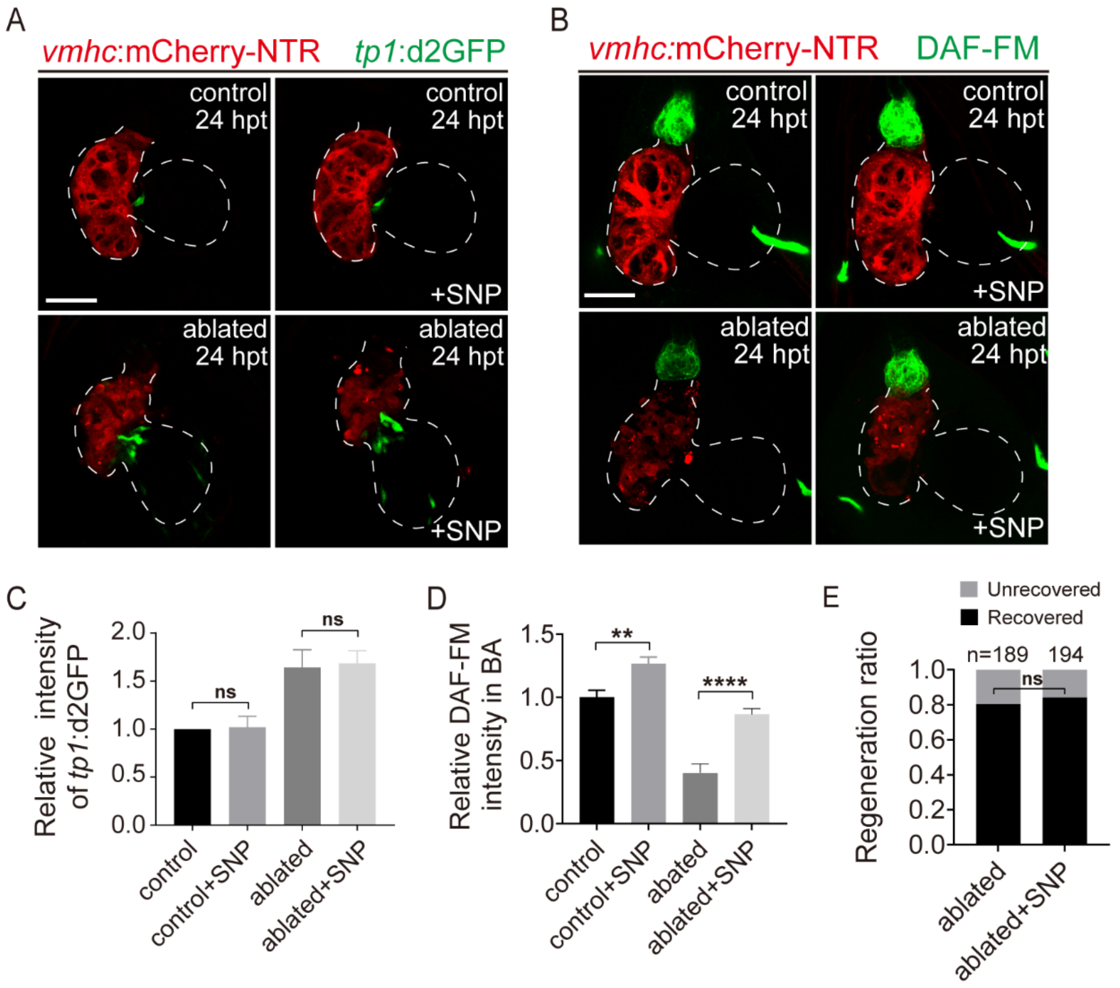

Supplementary Figure 8. NO supplement in the early stage of regeneration does not affect Notch signaling activation and heart regeneration

(A) Confocal images of *Tg(vmhc:mCherry-NTR; tp1:d2GFP)* control or ablated hearts with or without SNP treatment for 0-24 hpt.

(B) DAF-FM DA staining of *Tg(vmhc:mCherry-NTR)* control or ablated hearts with or without SNP treatment for 0-24 hpt.

(C) Quantification of relative *tp1*:d2GFP intensity in control, control + SNP, ablated and ablated + SNP hearts. N = 7 for each group. Mean + s.e.m. Student's t-test; ns, not significant.

(D) Quantification of relative DAF-FM DA intensity in the BA of control, control + SNP, ablated and ablated + SNP hearts. N = 7 for each group. Mean + s.e.m. Student's t-test; $**P < 0.01$, $****P < 0.0001$.

(E) Quantification of the heart recovery rate in ablated and ablated + SNP groups. The

numbers of larvae analyzed for each condition are indicated. Binomial test; ns, not significant.

Scale bars, 50 μm. Dashed lines outline the hearts. hpt, hours post treatment; NO, nitric oxide; SNP, sodium nitroprusside dihydrate.

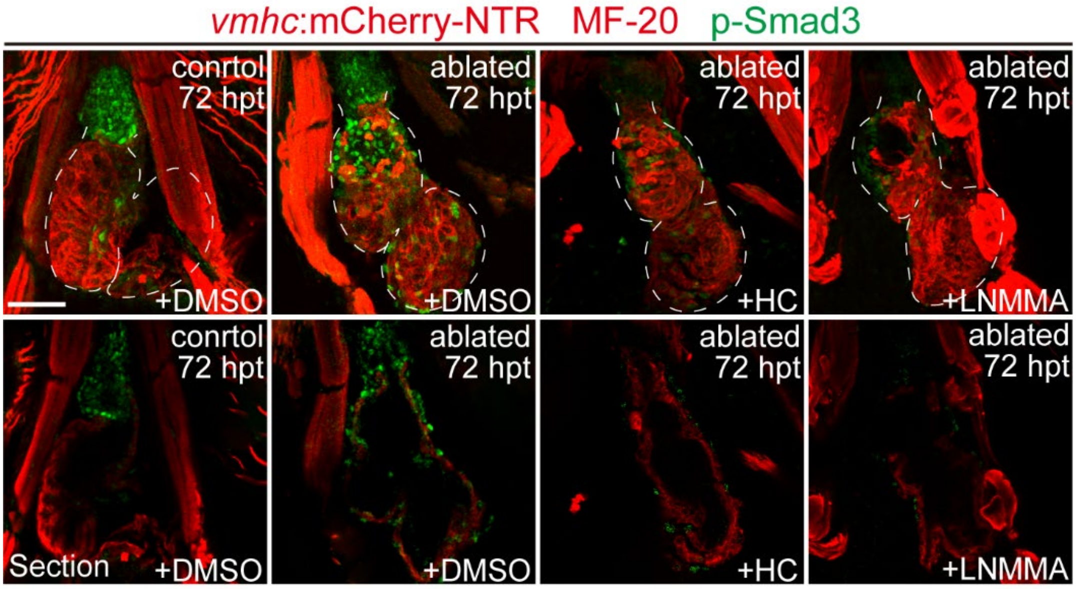

Supplementary Figure 9. TGF-β signaling is mainly activated in the epicardium and myocardium in ablated hearts

Immunostaining of anti-myosin heavy chain (MF-20, red) and anti-phospho-Smad3 (green) in control and ablated *Tg(vmhc:mCherry-NTR)* hearts treated with DMSO, HC-067047 or L-NMMA for 48-72 hpt. Image of single optical section revealed that TGF-β signaling was mainly activated in the epicardium and myocardium in ablated hearts. Scale bar, 50 μm. Dashed lines outline the hearts. hpt, hours post treatment; HC, HC-067047.

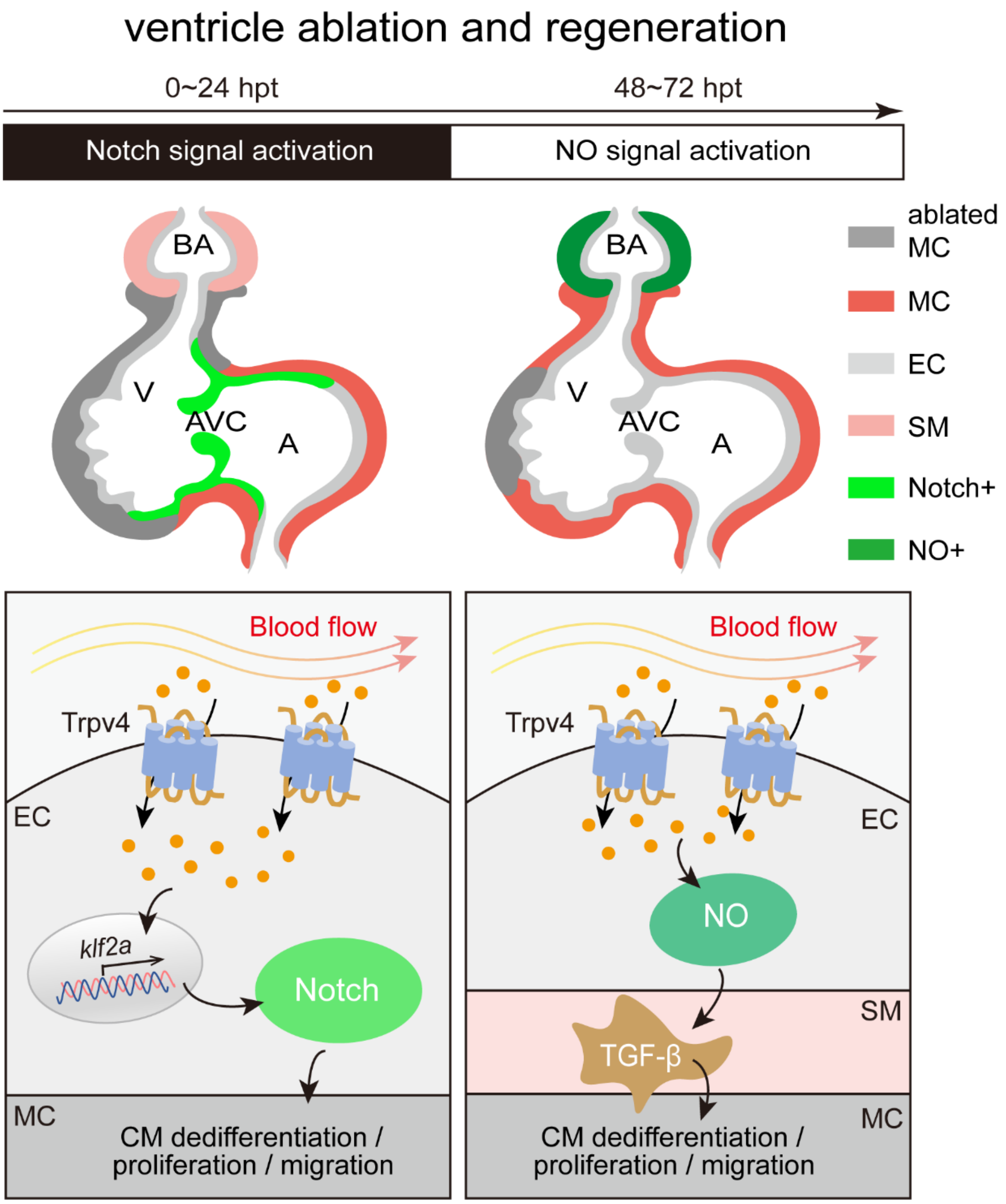

Supplementary Figure 10. Schematic diagram of spatiotemporal modulation of NO and Notch signaling by hemodynamic-responsive Trpv4 during ventricle regeneration

A, atrium; AVC, atrioventricular canal; BA, bulbus arteriosus; V, ventricle; NO, nitric oxide; MC, myocardium; EC, endocardium; SM, smooth muscle; CM, cardiomyocyte.

Supplementary Table 1. List of primers used in this study

Gene	primer (5'-3')	
**Real Time qPCR primers**		
trpv4	GGGGATGAACCCCCTCTATT	CAACAGCGACACTAATGCTGA
nos1	GCCAGAGGGTCATCGATAGGAA	TTGTTAAGCACCGTGGGCATG
nos2a	TGGGAAGACAAGCACAAACCAC	CTTAGCCGCTTTGTGATGAAGTGA
nos2b	GGTTGTTTGCATGGAGGACT	CTCCAGCACCTCAAGGAAAG
actb1	GATCAAGATCATTGCTCCCC	GGCCATTTAAGGTGGCAACA
**Semi-quantitative RT-PCR primers**		
trpv4-1	AGCAAGATTGAGAATCGGCA	CCACCACCAACACAGAGTAG
trpv4-2	GAACCCCCTCTATTTTACCAGA	AGTAGTCGCCCACTGCAA
actb1	CAGCCTTCCTTCCTGGGTAT	GCCATACAGAGCAGAAGCCA
**sgRNA PCR primers**		
trpv4	gatcactaatacgactcactataGGTGTCCATGCGCAGGCCAGgttttagagctagaa	
nos1-1	gatcactaatacgactcactataGGGAGGCTGTCCGGGAGACTgttttagagctagaa	
nos1-2	gatcactaatacgactcactataGGGAGGCTGTCCGGGAGACTgttttagagctagaa	
nos2a-1	gatcactaatacgactcactataGGGCCGCGGATCAGAGGTTTgttttagagctagaa	
nos2a-2	gatcactaatacgactcactataGGGGTCAGGGAACCAGACATgttttagagctagaa	
nos2b-1	gatcactaatacgactcactataGGGTTGGTATGCATTGCCTGgttttagagctagaa	
nos2b-2	gatcactaatacgactcactataGGGGAACTCACCTGGATTAGgttttagagctagaa	
tracRNA	AAAAAAAGCACCGACTCGGTGCCACT	
